# Biochemical Mechanisms of Sirtuin-Directed Protein Acylation in Hepatic Pathologies of Mitochondrial Dysfunction

**DOI:** 10.3390/cells11132045

**Published:** 2022-06-28

**Authors:** Courtney D. McGinnis, Erin Q. Jennings, Peter S. Harris, James J. Galligan, Kristofer S. Fritz

**Affiliations:** 1 Skaggs School of Pharmacy and Pharmaceutical Sciences, University of Colorado Anschutz Medical Campus, Aurora, CO 80045, USA; courtney.mcginnis@cuanschutz.edu (C.D.M.); peter.harris@cuanschutz.edu (P.S.H.); 2 Department of Pharmacology and Toxicology, College of Pharmacy, University of Arizona, Tucson, AZ 85721, USA; ejennings@pharmacy.arizona.edu (E.Q.J.); jgalligan@pharmacy.arizona.edu (J.J.G.)

**Keywords:** acetylation, sirtuin, histone deacetylase, mitochondria, non-alcoholic liver disease, metabolic syndrome, alcoholic liver disease

## Abstract

Mitochondrial protein acetylation is associated with a host of diseases including cancer, Alzheimer’s, and metabolic syndrome. Deciphering the mechanisms regarding how protein acetylation contributes to disease pathologies remains difficult due to the complex diversity of pathways targeted by lysine acetylation. Specifically, protein acetylation is thought to direct feedback from metabolism, whereby nutritional status influences mitochondrial pathways including beta-oxidation, the citric acid cycle, and the electron transport chain. Acetylation provides a crucial connection between hepatic metabolism and mitochondrial function. Dysregulation of protein acetylation throughout the cell can alter mitochondrial function and is associated with numerous liver diseases, including non-alcoholic and alcoholic fatty liver disease, steatohepatitis, and hepatocellular carcinoma. This review introduces biochemical mechanisms of protein acetylation in the regulation of mitochondrial function and hepatic diseases and offers a viewpoint on the potential for targeted therapies.

## 1. Introduction

The global burden of liver disease contributes to 2-4% of mortality, representing over 2 million deaths per year [1,2]. Among the many hepatic pathologies that result in end-stage liver disease (ESLD), cirrhosis (11th) and cancer (16th) were in the top 20 leading causes of death in 2018 and incidences of both continue to rise worldwide [1]. The prevalence and burden of ESLD presents a severe healthcare and therapeutic challenge, as the biochemical etiology is multifactorial and derived from altered metabolic signaling, excess triglyceride accumulation, mitochondrial dysregulation, oxidative stress, and inflammation [3]. Properly functioning mitochondrial metabolism is central to maintaining hepatocyte homeostasis and overall health [4].

Mitochondria are central organelles in hepatic nutrient disposition and metabolic pathways, including the citric acid cycle (TCA), beta-oxidation, and the electron transport chain (ETC). The disrupted synthesis and utilization of biochemical co-factors that serve as regulatory feedback systems to direct protein activity is a hallmark of altered mitochondrial metabolism [5]. While these processes are quite complex, nutrient-derived protein post-translational modifications (PTMs) are critical mediators in sensing and restoring metabolic flux. PTMs regulate protein function by altering amino acid characteristics like charge and shape to affect substrate and/or cofactor binding, dimerization, and subcellular localization. For example, charge-state disruptions due to lysine acetylation provides a distinct link between nutrient status and protein regulation, reflecting a snapshot of metabolic stress through the central metabolite acetyl-CoA (Ac-CoA) [6,7]. Mitochondria play a vital role in cellular energetics through the generation of Ac-CoA and adenosine triphosphate (ATP) to the cell through oxidative metabolism (i.e., the TCA cycle) [8]. These metabolites also serve as the primary substrates that facilitate protein phosphorylation (ATP) and acetylation (Ac-CoA), responding to changes in nutrient availability and redox state, thereby expanding the functional proteome [9]. Due to this central role, these PTMs are widespread, with over 60% of mitochondrial proteins being reported as acetylated, often at multiple Lys residues [10]. The variability in energy required by different tissues generates a mitochondrial acetylome landscape that can vary drastically between organs and even within regions of a single tissue [11,12].

Here, we present a review of protein acetylation known to impact mitochondrial function associated with altered hepatic metabolic states and disease pathogenesis. The last two decades have witnessed an expansion of descriptive and mechanistic insight into the regulatory nature of metabolism-derived PTMs [5]. This information is presented with a focus on mitochondrial homeostasis and the contribution of protein acetylation to hepatic pathologies. 

## 2. Biochemical Origins and Dynamics of Lys Acetylation

To survive environmental pressures, cells must be able to sense and react to changes in their environment, both endogenously and exogenously [5,13]. This is often achieved through PTMs that reflect nutrient availability and modulate feedback required to maintain cell growth and homeostasis [14,15]. The last universal common ancestor likely evolved through pressures placed on the cell and these adaptations were achieved through conserved PTMs (i.e., phosphorylation, glycosylation, acetylation) that are now found ubiquitously across all life [5,13]. For example, the primary substrate for Lys acetylation, Ac-CoA, may have originated through the Wood-Ljungdahl carbon-fixing pathway in eubacteria and archaebacteria [16]. Biochemical adaptations over time contributed to the diversity of cellular protein regulation, as currently over 700 known PTMs are listed in the Uniprot PTM Knowledgebase [13,17]. Ac-CoA persists as a key metabolic substrate and co-factor central in higher forms of life, thus evolving a role for protein regulation through Lys acetylation [16].

**Figure 1:**
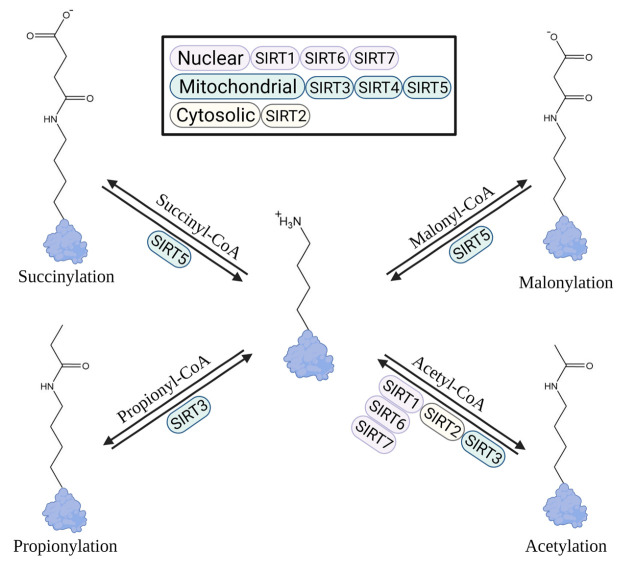
Acyl-CoA species generated during central metabolism modify protein Lys residues. Deacylases (sirtuins) remove these Lys modifications. Acetyl-CoA is removed by SIRT1 in the nucleus and cytosol, SIRT6 and SIRT7 in the nucleus, SIRT2 in the cytosol and nucleus, and SIRT3 in the mitochondria. Malonyl-CoA and succinyl-CoA are both removed by mitochondrial SIRT5 [28]. Propionylation is removed by mitochondrial SIRT3 [35]. Abbreviations: SIRT, sirtuin.

As a regulator of metabolic homeostasis, Lys acetylation is tightly regulated via enzymatic addition and/or removal [5]. Site-specificity arises from local physiochemical properties that govern the susceptibility of a Lys residue to modification, including pH, electrostatics, substrate, and co-factor binding [18]. Enzymatically derived acetylation is achieved through the acetyl transfer from Ac-CoA to a Lys by a dedicated writer protein (e.g., Lys acetyltransferase, KAT; histone acetyltransferase, HAT) [5,19]. Enzymatic acetylation is notably focused toward cytosolic and nuclear processes, such as histone acetylation and the regulation of gene expression. In contrast, non-enzymatic modification by acyl-CoA’s occurs throughout the cell on Lys and Cys via a non-enzymatic acyl transfer of the thiol-linked moiety to the ε-amine or N-terminus of Lys residues or the thiol group of Cys residues [20,21]. This non-enzymatic mechanism is recognized as a major source of protein acetylation within the mitochondria. Indeed, just as acetyl-CoA leads to Lys acetylation numerous other acyl-CoA metabolites contribute to lysine modification, thus the term “acylation” due to numerous acyl-CoA species. In addition, NAD^+^/NADH (nicotinamide adenine dinucleotide) and NADP^+^/NADPH (nicotinamide adenine dinucleotide phosphate) within the cytosol and mitochondria may play a role in acyl-CoA reactivity toward protein Lys residues through the regulation of subcellular pH [22]. While Ac-CoA is most prominent in the liver, other acyl-CoA species such as succinyl-CoA, glutaryl-CoA, and propionyl-CoA play a significant regulatory role on protein function [5,23,24]. Indeed, these acyl-CoA species alter protein activity through different mechanisms. For example, Lys acetylation renders the positive charge of Lys neutral while Lys succinylation induces a negative charge on the modified Lys (Figure 1). Aldh2 activity can be inhibited in this way through the acetylation of Lys369 which prevents the binding of a necessary cofactor, NAD^+^ [25]. Altogether, these subtle yet distinct charge differences, along with size and shape, induce a complex regulatory response throughout the mitochondrial proteome that remains poorly characterized [26,27,28,29]. 

In the mitochondria, Class III histone deacetylases (HDACs), or sirtuins (SIRTs), perform the bulk of Lys deacylation [30]. Sirtuins (SIRT1-7) are NAD^+^ dependent deacylases broadly defined by their subcellular localization [30,31]. These eraser enzymes use NAD^+^ to remove acyl moieties from Lys residues, yielding an unmodified Lys and 2’-*O*-acyl-adenosine diphosphate ribose [30,31]. Zinc-binding Cys residues in the small subunit on the outside of the catalytic pocket also allows sirtuins to act as metabolic redox sensors [32,33]. SIRT3, 4, and 5 are predominately localized to the mitochondria through an N-terminal mitochondrial localization sequence that is cleaved upon entry into the mitochondria [34]. SIRT3 is believed to be the dominant Lys deacetylase, with a reduced capacity to remove Lys propionylation [35]. While SIRT4 displays very little deacetylase activity, there is evidence that it can remove lipoylation, biotinylation, methylglutarylation, and mono-ADP-ribosylation [36]. SIRT5 also shows significantly reduced deacetylase activity when compared to SIRT1-3; however, SIRT5 removes multiple acylations that stem from mitochondrial CoA precursors, such as succinyl, malonyl, and glutarylLys [28,29,37]. Despite being in the nucleus, SIRT6 contributes to the maintenance of mitochondrial processes through its activity as a histone deacetylase and a few examples are given below [38,39]. Lastly, SIRT7 resides in the nucleus and cytosol and has been shown to remove acetyl and myristoyl groups from Lys residues to support mitochondrial metabolic processes (Figure 1) [40]. Each passing year reveals novel sirtuin substrates, mainly derived from metabolically linked CoA species, like the recently described lactoyl-CoA induced Lys lactoylation [20,41]. While several studies exist that define key roles for sirtuins in cellular redox control and metabolic regulation, further research is needed to wholly uncover regulatory mechanisms that may contribute to numerous hepatic pathologies.

## 3. Hepatic Metabolism and Protein Acylation

**Figure 2:**
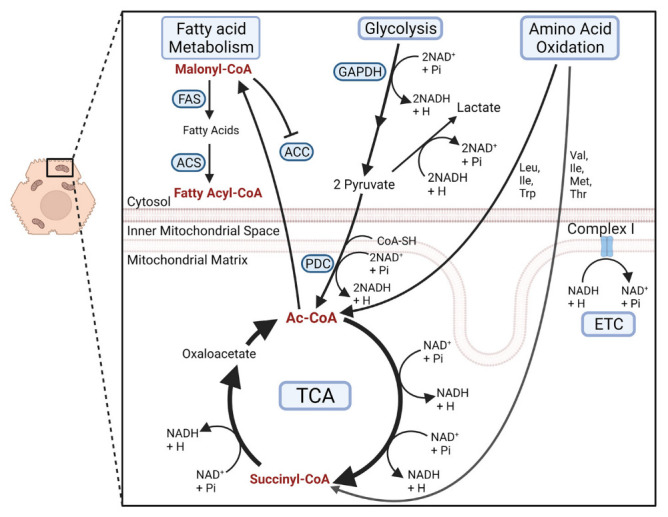
Reactive acyl groups are generated through the citric acid cycle (TCA), fatty acid metabolism, glycolysis, and amino acid oxidation. Additionally, nicotinamide adenine dinucleotide (NAD^+^/NADH) redox ratios are controlled by different metabolic processes as electron shuttling molecules. Abbreviations: ACC, acetyl-CoA carboxylase; Ac-CoA, acetyl-CoA; ACS, acyl-CoA synthase; ETC, electron transport chain; FAS, fatty acid synthase; GAPDH, glyceraldehyde 3-phosphate dehydrogenase; Ile, isoleucine; H, hydrogen; Leu, leucine; Met, methionine; NADH, nicotinamide adenine dinucleotide; PDC, pyruvate dehydrogenase complex; Pi, phosphate group; TCA, citric acid cycle; Thr, threonine; Trp, tryptophan; Val, valine.

The liver is largely responsible for filtering blood exiting the digestive tract. This central function results in the uptake of proteins, lipids, and carbohydrates into hepatocytes. Thus, the liver has adapted many dynamic metabolic pathways to accommodate dietary fluctuations including glycolysis, lipogenesis, ketogenesis, triglyceride synthesis, gluconeogenesis, and lipid metabolism [42]. Each of these primary metabolic pathways generate key intermediates that serve as precursors for mitochondrial PTMs [5,6,43]. Dietary carbohydrates are broken down by the digestive system, converted to glucose, and taken up into hepatocytes via glucose transporter 2 (GLUT2). Glucose is then converted to pyruvate and subsequently Ac-CoA, which enters the TCA cycle [44,45]. During nutrient intake, Ac-CoA is shuttled to the cytosol where it is carboxylated by Ac-CoA carboxylase (ACC) to generate malonyl-CoA. During fed states, malonyl-CoA concentrations increase, inhibiting ACC and favoring *de novo* lipogenesis. Fatty acid synthase (FAS) uses malonyl-CoA to generate fatty acids; acyl-CoA synthase (ACS) then converts these fatty acids to fatty-acyl-CoAs (Figure 2) [43]. As acyl-CoA metabolite abundance increases in cytosolic and mitochondrial compartments, they induce the acylation of Lys residues to modify protein structure and function in response to cellular metabolism. 

**Figure 3:**
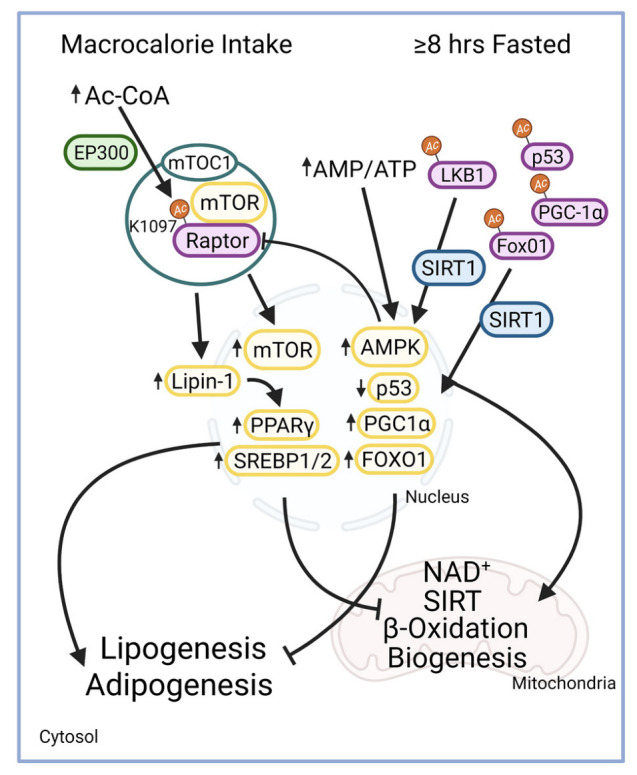
mTOR and AMPK respond to fasted and fed states. During a fed state, increased Ac-CoA is observed along with decreased NAD^+^ resulting in reduced SIRT activity. mTORC1 is regulated through acetylation of Raptor by EP300, an acetyltransferase, subsequently inducing mTOR signaling [61]. This results in the phosphorylation of lipin-1 and the activation of lipogenic and adipogenic transcription factors SREBP1/2 and PPAR-γ [46]. A fasted state occurs in hepatocytes after 8–12 h of restricted caloric intake resulting in the activation of AMPK by two mechanisms, increased AMP/ATP ratio and LKB1 phosphorylation [48]. SIRT1 activity is altered through AMPK-enhanced NAD^+^ concentrations, thus enabling SIRT1 to deacetylate LKB1 and activating AMPK [54]. SIRT1 also regulates p53, PGC-1α, and FoxO1 to promote mitochondrial biogenesis [49,55]. Abbreviations: Ac-CoA, acetyl-CoA; AMP, adenosine monophosphate; AMPK, adenosine monophosphate-activated protein kinase; ATP, adenosine triphosphate; FAS, fatty acid synthase; FoxO1, forkhead box1; LKB1, liver kinase beta 1; mTOR, mammalian target of rapamycin; mTORC1, mTOR complex 1; NAD^+^, nicotinamide adenine dinucleotide; PGC-1α, proliferator-activated receptors and γ coactivator 1α; PPAR-γ, peroxisome proliferator-activated receptor gamma; SIRT, sirtuin; SREBP1, sterol regulatory element binding protein 1.

Maintaining homeostasis between fed and fasted states is dependent on metabolite availability and signaling pathway activation. mTOR (mammalian target of rapamycin) and AMPK (adenosine monophosphate-activated protein kinase) are two important regulatory pathways that are activated during fed and fasted states, respectively [46,47]. mTOR is a subunit of two complexes, mTOR complex 1 (mTORC1) and mTOR complex 2 (mTORC2). mTORC1 is activated by EP300-dependent acetylation of Raptor (regulatory-associated protein of mammalian target rapamycin), a core component of the mTORC1 complex. mTORC1 activation promotes lipid synthesis, cell growth, autophagy suppression, and mitochondrial fission through multiple avenues: the phosphorylation of lipin-1 and the activation of transcription factors, PPARγ, and SREBP1 [48,49,50]. When a fed state is prolonged, it may disrupt mitochondrial function through diminished beta-oxidation and increased mitochondrial fission and lipid formation (Figure 3) [46,51,52]. 

Recent evidence suggests a link between cellular NAD^+^ levels, sirtuin activity, and the regulatory factors described above. AMPK has been found to increase NAD^+^ concentrations, as a metabolic sensor activated during fasted states by the increased AMP (adenosine monophosphate) to ATP ratio [53]. Additionally, SIRT1 has been found to contribute to the activation of AMPK via deacetylation of liver kinase beta 1 (LKB1), an AMPK kinase, supporting mitochondrial processes and generating a positive feedback loop [48,53,54]. SIRT1 is also a crucial regulator of several transcription factors: p53, PPAR, PGC-1α, and FoxO1 through Lys deacetylation. Each of these has been associated with the expression of genes in mitochondrial and lipid metabolism [49,54]. Specifically, SIRT1 deacetylates FoxO1 at Lys242, Lys245, and Lys262 and FoxO3 at Lys242, Lys259, Lys271, Lys290 and Lys569 [50,55,56]. The interaction of AMPK and sirtuins provides an adaptive mechanism associated with mitochondrial beta-oxidation, autophagy, mitochondrial biogenesis, mitophagy, and mitochondrial fusion in response to dietary changes (Figure 3) [53,57,58,59,60]. Together, mTOR and AMPK respond to changes in nutrient states and mediate the effects of reactive acyl groups with numerous direct and indirect consequences on mitochondrial function [16].

## 4. Protein Acetylation and Mitochondrial Dysfunction in Hepatic Pathologies

Acyl-CoA species and associated PTMs regulate mitochondrial metabolism, biogenesis, and cellular redox with broad implications for overall hepatocyte function. Maintenance of mitochondrial homeostasis is regulated, in part, through protein acylation. Disruption of a single protein or pathway may trigger a cascade of deleterious signaling events, yet few examples as well-defined as pyruvate kinase M2 (PKM2) acetylation exist [62,63]. The sections below describe pathological observations that are associated with altered protein acetylation in rodent and cell culture models resulting from various metabolic derangements. Key studies emphasizing the therapeutic potential of sirtuin activity are also described. While no single acetylation event has been identified as a cause for liver disease or dysfunction, strong evidence supports the accumulation of acetylLys on metabolic and antioxidant proteins as a pathogenic mechanism in hepatic diseases.

### 4.1. Non-Alcoholic Fatty Liver Disease

Non-alcoholic fatty liver disease (NAFLD) is defined as the accumulation of fat within the liver exceeding 5 to 10% [64]. This disease affects 24% of adults or an estimated 62 million people in the United States [64]. More progressive forms of NAFLD, such as non-alcoholic steatohepatitis (NASH), affect up to 6.5% of the U.S. adult population [65]. Unfortunately, NAFLD has recently been observed in teenagers with an estimated prevalence as high as 17% [66]. The progression of NAFLD is associated with type 2 diabetes (T2D) and obesity, leading to the accumulation of hepatic triglycerides and free fatty acids [67,68]. A high-fat diet (HFD), sustained consumption of fructose, and a sedentary lifestyle are driving factors that contribute to NAFLD [69,70]. 

Mitochondrial dysfunction and oxidative stress are hallmarks of NAFLD. While numerous factors contribute to mitochondrial dysfunction, recent evidence demonstrates a significant decrease in mitochondrial SIRT3 activity in murine models of NAFLD [71,72]. This decrease in SIRT3 activity correlates with increased mitochondrial protein acetylation. The findings included 193 preferentially acetylated proteins in HFD-fed mice versus controls, with many of the protein targets involved in gluconeogenesis, mitochondrial oxidative metabolism, methionine metabolism, liver injury, and the endoplasmic reticulum (ER) stress response [71].

Recent studies have found an association with the decrease in SIRT3 activity and hepatic steatosis [73]. SIRT3 deacetylates long-chain acyl-CoA dehydrogenase (LCAD), the enzyme responsible for initiating mitochondrial beta-oxidation. The acetylation of LCAD decreases enzyme activity and results in reduced beta-oxidation [73,74,75,76]. When fed a HFD, Sirt3 knockout (KO) mice develop hepatic steatosis and have diminished beta-oxidation gene activation compared to wild-type (WT) controls. Furthermore, KO mice have reduced expression of *Ppar*α, along with gene targets critical to fatty acid uptake (*CPT-1a*) and beta oxidation (*Mcad*). Additionally, this study found multiple Nrf2 (nuclear factor (erythroid-derived 2)-like 2) regulated genes were elevated in SIRT3 KO mice, such as very low-density lipoprotein receptor (*Vldlr*) and *Cd36* [77]. *Vldlr* binds intermediate density lipoproteins letting lipids enter the cell and CD36 is a fatty acid transporter; both *Vldlr* and *Cd36* contribute to triglyceride accumulation [77]. Conversely, a recent article found that in mice, liver specific Sirt3 overexpression did not provide whole-body protection against triglyceride accumulation nor insulin resistance [78]. The discrepancy of SIRT3’s role displays the importance to further investigate the role of SIRT3. 

Both SIRT1 and SIRT3 deacetylate acyl-CoA synthase 1 and 2 (ACS), respectively, at Lys642, demonstrating a coordinated effort by SIRTs to regulate metabolism across cytosolic and mitochondrial subcellular compartments [79]. Sirtuin directed deacetylation activates ACS which catalyzes the transformation of acetate to Ac-CoA [79,80]. SIRT1 and SIRT3 are also heavily involved in ketogenesis through the deacetylation of 3-hydroxy-3-methylglutaryl CoA (HGM-CoA) synthase 1 and 2, respectively, at Lys310 [80]. The regulation of ketogenesis and fatty acid metabolism supports the importance of non-mitochondrial sirtuin activity in the maintenance of mitochondrial homeostasis and oxidative stress. During the progression of NAFLD to NASH a drastic increase in the accumulation of reactive oxygen species (ROS) plays a role in the initiation of inflammatory responses [81]. Mitochondrial ROS are generated through the ETC and alpha-ketoglutarate dehydrogenase, among other pathways [81]. SIRT3 contributes to the mitochondrial ROS pool through the regulation of superoxide dismutase (SOD2) deacetylation at Lys68 and Lys122. Here, an increase in the acetylation of SOD2 inhibited enzyme activity and led to an increase in ROS, contributing to the pathogenesis of NASH [82,83,84]. 

One important enzyme to the development and progression of NAFLD is peroxisomal acyl-CoA oxidase 1 (ACOX1). ACOX1 initiates beta-oxidation by breaking down acyl-CoAs to 2-trans-enoyl-CoA. A study investigating NAFLD progression utilized a HFD and an ACOX1 KO mouse model to investigate the disadvantageous role of ACOX1 during the development of steatosis [51]. The loss of ACOX1 increased mitochondrial beta-oxidation, which was determined using labeled palmitate to enable the researcher to track the metabolism of fatty acids. This study also revealed that ACOX1 drove the inhibition of autophagy and lipophagy through the activation of mTOR by elevated Ac-CoA in the peroxisome [51]. Alongside mTOR activation, Lys acetylation regulates several transcription factors that play a role in nuclear, cytosolic, and mitochondrial signaling. One example is the activation of mTOR, which occurs via the acetylation of RAPTOR to enhance the accumulation of fatty acids and contributes to the development of steatosis [51,85]. mTOR activates SREBP1/2, a transcription factor that regulates several genes associated with lipogenesis and cholesterol maintenance [acyl-CoA carboxylase1 (ACC), fatty acid synthase (FASN), and low-density receptor gene (LDLR)] [86]. Interestingly, LB100, a serine/threonine protein phosphatase 2A (PP2A) small molecule inhibitor, demonstrated inhibition of PP2A, leading to increased SIRT1 expression and AMPK phosphorylation. When treated with LB100, a decrease in both SREBP1 and FAS abundance was observed when applied to a HFD model. Overall, LB100 treatment improved liver health in the HFD treatment group with decreased accumulation of lipid, alanine aminotransferases (ALT), aspartate aminotransferases (AST), and fasting insulin [87]. Another transcription factor associated with the development of NAFLD, cyclic AMP-responsive element-binding protein 3-like-3-hepatocyte-specific (CREBH), was found to be regulated by SIRT1 under fasting conditions [88,89]. The authors found that acetylation of CREBH at Lys294 is required for transcriptional activation critical to the regulation of lipolysis, beta-oxidation, and ketogenesis. Lastly, the transcription factor carbohydrate-responsive element–binding protein (ChREBP) has also been connected to NAFLD as a regulator of glycolytic and lipogenic genes and is activated by the acetylation of Lys672 by EP300 [90,91].

Apart from a well-balanced diet, exercise, and a reduction in caloric intake, many therapies are being investigated to ameliorate the progression of NAFLD; however, no effective treatments have been found to date. One area of therapeutic investigation for NAFLD has focused on NAD^+^ concentrations in NAFLD phenotypes in both animal models and clinical settings [92,93]. Nicotinamide phophoribosyltransferase (NAMPT), which converts nicotinamide to NAD^+^, is decreased in patients with NAFLD and NASH compared to healthy controls [94,95]. Based on these observations, NAMPT-targeted inhibitors have been developed for further mechanistic investigation, such as FK866, which enhanced steatosis and increased expression of SREBP1 and FAS in a HFD murine model [96]. Studies targeting sirtuins directly (competitive inhibitors) or indirectly (NAD^+^ boosting) have shown that activation of SIRTs may be a double-edged sword, demonstrating positive and negative effects regarding liver health. Thus, therapies should be tailored to specific metabolic circumstances as treatment developed for NAFLD may prove deleterious in situations of hepatocellular carcinoma (HCC) [97,98]. Additionally, NAFLD has been identified as a risk factor for cholangiocarcinoma (CCA), a biliary malignancy arising from cholangiocytes [99]. Recent studies have found histone acetylation contributes to CCA pathogenesis and HDAC modulators are promising for treatment, and this topic has been extensively review by Pant et al [100]. The SIRT1 targeting resveratrol was proposed to improve outcomes of patients with NAFLD by increasing weight loss and decreasing triglycerides and leptin/adiponectin ratio, but no significant improvement was observed in liver enzymes [101,102,103,104,105]. Additionally, the SIRT1 activator coumetarol induces mitochondrial biogenesis through increased intracellular NAD^+^/NADH ratios [106]. Regarding mitochondrial health, berberine treatment increases SIRT3 expression and improves patient outcomes through increased beta-oxidation and decrease ROS production [107,108]. Similar effects have been observed using dihydromyricetin, increasing mitochondrial respiratory capacity and redox homeostasis through AMPK and SOD2 activation [109,110]. Recent studies examined the effects of hydrogen sulfate (H_2_S) as a novel gastrotransmitter to decrease the accumulation of hepatic Ac-CoA and lipids. Mechanistically, H_2_S increases PPAR expression and activates ACOX1, promoting beta-oxidation and the prevention of steatosis [111]. Further studies are still needed to define interventions targeting lipid metabolism and mitochondrial homeostasis through protein acetylation in models of NAFLD.

### 4.2. Metabolic Syndrome

Metabolic syndrome (MetS) is defined by key characteristics that result in an increased incidence of cardiovascular disease, stroke, diabetes, and other chronic diseases [112]. These include obesity, hyperlipidemia, hypoalphalipoproteinemia, hypertension, and hyperglycemia with insulin resistance. Unfortunately, the development of MetS has become a worldwide problem [113,114]. The prevalence of US adults (18 and older) with MetS was 38.3% in 2018, representing 95 million Americans [115,116]. 

The accumulation of abdominal fat is a critical factor in the development of MetS. An analysis of 29 severely obese patients associated with metabolic syndrome found that weight loss resulted in the activation of SIRT1, SIRT3, and SIRT6 expression in hepatic and adipose tissue [117]. Indeed, decreased SIRT6 expression and increased protein acetylation is associated with characteristics of pre-diabetic overweight patients, including higher levels of PPAR-γ and SREBP1 [118]. Another study revealed that SIRT6 overexpression significantly decreased cholesterol and triglyceride levels, increased rate-limiting enzymes in the liver to enhance beta-oxidation while also activating AMPK, providing support for an association between homeostatic glucose metabolism, mitochondrial metabolism, and SIRT6 [118,119]. Given the preponderance of MetS, multiple translational models have been developed—typically consisting of a long-term HFD or a Western diet [120]. In one rodent model of metabolic syndrome, hepatic SIRT6 was positively associated with SIRT1 and was found to improve blood lipid profiles to protect against adipose-induced oxidative stress [121]. These authors demonstrated that hepatic-specific deletion of SIRT6 resulted in increased blood glucose and hepatic steatosis, inducing metabolic syndrome. Aside from SIRT6, one chronic HFD model increased mitochondrial protein acetylation via increased lipid metabolism and decreased SIRT3 deacetylase activity [122]. The absence of SIRT3 led to hyperacetylation of LCAD at Lys318 and Lys322 which was found to contribute to a decrease in beta-oxidation and increased lipid accumulation, promoting MetS [73,75,76]. Conversely, fasting upregulates SIRT3 expression to improve LCAD activity and beta-oxidation, demonstrating the importance of SIRT3 deacetylase activity in the maintenance of mitochondrial function and overall hepatocyte health [76]. 

**Figure 4:**
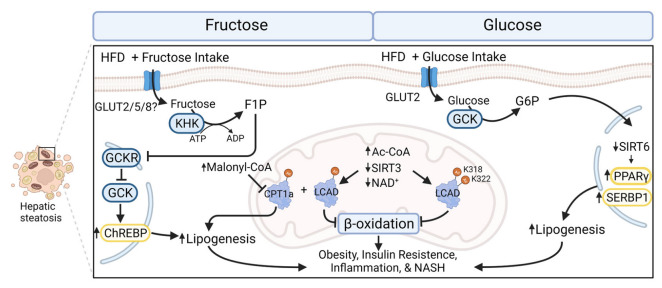
Fructose and glucose metabolism in combination with HFD are connected to negative health outcome consistent with MetS. Fructose upon cellular uptake by GLUT2, 5 and possibly 8, is catabolized to F1P by KHK using the cofactor ATP. In contrast, glucose is metabolized to G6P by GCK which is inhibited by GCKR [125]. KHK is not allosterically inhibited resulting in the excess generation of F1P in hepatocytes [126]. F1P can interfere with GCK inhibition by dislocating its binding partner GCKR. Resulting in the induction of the transcription factor ChREBP, resulting in increased lipogenesis [126]. Fructose consumption also induces hyperacetylation of CPT1a and LCAD which leads to decreased lipid metabolism [69]. Fructose metabolism is also connected to elevated concentrations of malonyl-CoA which inhibits CPT1a, reducing mitochondrial lipid uptake. Glucose consumption results in the acetylation of LCAD and decreased beta-oxidation [73]. Glucose consumption is also associated with the decreased SIRT6 abundance and increased lipogenesis [118]. Abbreviations: ac-CoA, acetyl-CoA; ChREBP, carbohydrate-responsive element–binding protein; CPT1a, carnitine palmitoyltransferase 1a; GCK, glucokinase; GCKR, glucokinase regulatory protein; GLUT2/5/8, glucose transporter 2/5/8; LCAD, long-chain acyl-CoA dehydrogenase; NAD^+^, nicotinamide adenine dinucleotide; NASH, non-alcoholic steatohepatitis; PPAR-γ, proliferator-activated receptors and γ coactivator 1α; SIRT, sirtuin; SREBP1, sterol regulatory element binding protein 1.

Mitochondria respond dynamically to changes in metabolic intake and undergo increased stress due to fructose consumption when compared to glucose [123,124]. Fructose metabolism differs from glucose metabolism in several ways. Fructose is catabolized by ketohexokinase (KHK) to fructose-1-kinase (F1P) using the cofactor ATP. In contrast, glucose is metabolized to glucose-6-phosphate (GP6) by glucokinase (GCK) which is inhibited by GCKR (glucokinase regulatory protein) [125]. Unlike GCK, KHK is not allosterically inhibited by signals of cellular energy sufficiency or its product, resulting in rapid accumulation of F1P in hepatocytes [126]. Additionally, F1P can interfere with mitochondrial to nucleus signaling by interfering with GCK inhibition by dislocating its binding partner GCKR. This results in the induction of transcription factors like ChREBP, which has been connected to hepatic steatosis and lipid composition restructuring [126]. The stark differences between fructose and glucose metabolism are associated with impaired insulin signaling and the induction of obesity [127]. Interestingly, fructose consumption was also found to increase malonyl-CoA concentrations resulting in the inhibition of carnitine palmitoyltransferase 1a (CPT1a) and restricting the transport of lipids into the mitochondria, reducing beta-oxidation (Figure 4). Concurrent ingestion of a HFD and fructose increases hyperacetylation of CPT1a and LCAD, reducing the capacity for beta-oxidation. This diet also alters mitochondrial morphology, resulting in elevated mitochondrial number and decreased size [128]. 

Given the complex pathological development of MetS, several approaches for treatment have been explored to moderate the onset of severe disease. Recent evidence strongly supports the hypothesis that increasing SIRT1 activity is protective against HFD-induced MetS [129]. Indeed, resveratrol activates SIRT1 to improve mitochondrial beta-oxidation and aerobic capacity via deacetylation of PGC-1α in mice fed a HFD [101,129,130]. Unfortunately, clinical trials employing resveratrol found little effect on the metabolic health of overweight patients or those with MetS [131,132]. Since the combined effects of T2D and obesity increase hepatic pathology in NAFLD, the use of nicotinamide riboside (NR), a precursor to NAD^+^, has been examined as a therapeutic in T2D models [133]. NR treatment protects hepatic NADP^+^ and NADPH levels, decreases body weight, reduces lipid droplet area, and improves hepatic steatosis and hyperglycemia [134]. NAD^+^ supplementation to boost sirtuin activity also improves overall hepatocellular function and hepatic health, as demonstrated in a murine model for NASH (Table 1) [95]. Alternative MetS treatments, such as time restricted feeding, are being studied for the potential to induce the expression of fasting related proteins such as sirtuins, AMPK, and FoxO1 [135,136]. Lastly, metformin mediates abdominal fat accumulation by increasing SIRT6 expression and reducing lipogenesis related transcription factors (Table 1) [118]. In all, MetS is a metabolically driven disease with deep connections to changes in mitochondrial protein function and signaling due to Ac-CoA modifications impacting mitochondrial stress and lipid accumulation.

### 4.3. Alcohol-Associated Liver Disease

Alcohol-associated liver disease (ALD) is a significant source of morbidity and mortality, contributing to approximately 3.3 million deaths every year worldwide [143]. ALD encompasses several pathological injuries due to the consumption of alcohol, including moderate to advanced forms such as alcohol-associated steatohepatitis (ASH), alcohol-associated hepatitis (AH), and alcohol-associated cirrhosis (AC). Those who chronically consume alcohol are prone to developing steatosis (fatty liver) and greater than 10% will develop ASH [143]. 

Ethanol is primarily metabolized by alcohol dehydrogenase (ADH) and cytochrome P450 family 2 subfamily E member 1 (CYP2E1) [144]. Both ADH and CYP2E1 catalyze the conversion of alcohol to acetaldehyde [144]. Acetaldehyde is further metabolized by mitochondrial aldehyde dehydrogenase (ALDH2) to acetate [145]. When the capacity for ALDH2 to metabolize acetaldehyde is saturated, this electrophilic metabolite accumulates and generates protein and DNA adducts, contributing to hepatocyte damage and liver dysfunction [146]. Alcohol metabolism contributes directly to mitochondrial protein hyperacetylation whereas sirtuin deacetylase activity reverses alcohol-induced acetylation [147,148,149,150,151,152]. The pathogenesis and progression of ALD is thought to occur through several mechanisms including oxidative stress, inflammation, and mitochondrial dysfunction. These hypotheses are supported using several well-described rodent models of ALD [153,154,155]. Collectively, these studies demonstrate varied levels of hepatic mitochondrial acetylation with a more robust measure of acetylation in chronic models compared to acute models [156]. Following six weeks of ethanol consumption, mitochondrial protein acetylation was found to be elevated 5-fold [150]. Additionally, a CYP2E1 KO rat was used to demonstrate that protein acetylation occurs independently of CYP2E1 activity, suggesting ADH and ALDH2 may be central to mechanisms of ethanol-induced acetylation [150]. After 7 days of alcohol withdrawal, protein acetylation is reduced by half, although this could either be due to sirtuin-mediated deacetylase activity and/or protein turnover [150]. Aside from an increase in protein acetylation, ethanol metabolism increases lysine propionylation while lysine succinylation decreases [157]. A proteomic inventory of murine acetylation targets in ALD identified an increase in the acetylation of 395 proteins. Functional enrichment analyses reveals a marked enrichment in enzymes associated with lipid metabolism, energy production, amino acid metabolism, carbohydrate metabolism, stress response, and oxidative stress [157]. Akin to models of NAFLD, acetylation of SOD2 is increased in ALD, likely mediating the observed reduction in activity. These findings demonstrate that alcohol metabolism increases mitochondrial protein acetylation and contributes to altered lipid metabolism and oxidative stress [158].

**Figure 5:**
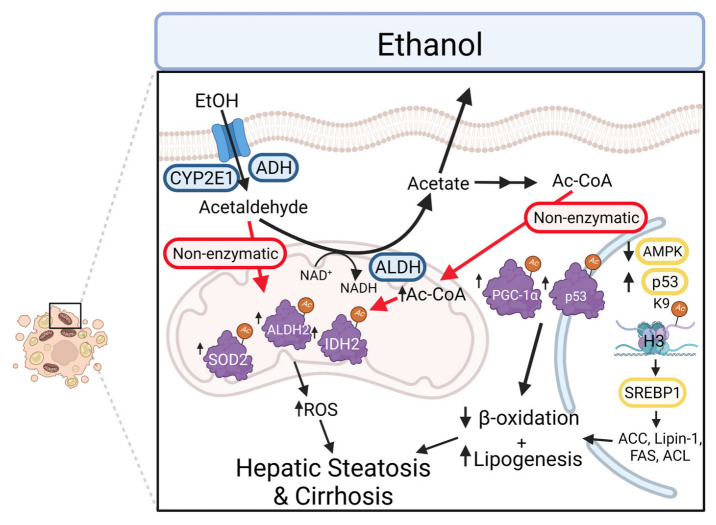
Alcohol metabolism by ADH, CYP2E1 and ALDH2 results in the generation of reactive metabolites acetaldehyde and acetate. The increased generation of acetaldehyde and Ac-CoA results in increased hepatic protein acetylation through non-enzymatic mechanisms. Increased acetylation affects specific proteins that are involved in mitochondrial health, such as SOD2, ALDH2, IDH2, PGC-1α, and p53 [147,149]. Additionally, changes to transcription factors are observed with a decrease in AMPK (inhibiting autophagy) and increase in p53 (correlated with cell death), contributing to the development of cirrhosis [147]. The acetylation of histone H3 has been associated with SREBP1 and an increased expression of lipogenic proteins, contributing to hepatic steatosis [161,163]. Abbreviations: ACC, acetyl-CoA carboxylase; Ac-CoA, acetyl-CoA; ADH, alcohol dehydrogenase; ALDH2, aldehyde dehydrogenase 2; AMPK, adenosine monophosphate-activated protein kinase; CYP2E1, cytochrome P450 family 2 subfamily E member 1; FAS, fatty acid synthase; IDH2, isocitrate dehydrogenase 2; PGC-1α, proliferator-activated receptors and γ coactivator 1α; SOD2, superoxide dismutase.

Alcohol-induced hyperacetylation impacts protein pathways significantly associated with decreases in NAD^+^ concentrations and sirtuin activity (Figure 5). Both p53 and PGC-1α have been found to be acetylated in chronic alcohol models and FOXO has been found to be deactivated, demonstrating that ethanol disrupts mitochondrial-nuclear signaling, contributing to mitochondrial dysfunction [149,159]. SIRT1 plays an important regulatory role in the activation of FOXO and p53, where acetylation activates p53 and diminishes mitochondrial respiration through cytochrome c. p53 was found hyperacetylated in this model, suggesting a link between ethanol-induced acetylation of p53 and mitochondrial dysfunction. These results demonstrate that the deacetylation of p53 by SIRT1 is a protective mechanism in ALD [160]. This study also revealed that SIRT5 expression was decreased, leading to the acetylation of certain mitochondrial proteins [149]. SIRT1 also regulates PPAR, SREBP1, NF-κB, and AMPK, all of which play an important role in ALD pathology through steatosis and indirect effects on mitochondrial function [161,162].

Aside from mitochondrial protein acetylation, histones and cytoskeletal proteins are known targets of alcohol-induced acetylation [164]. For example, one study found an increase in the acetylation of histone H3 due to alcohol metabolism and the acetylation of histone H3 at lysine 9 (H3K9ac) is known to impact AMPK/SREBP1 signaling [162,164,165,166]. Interestingly, acetylation of microtubules results in impaired transcellular transport of proteins and vesicle movement; lipid droplets are typically motile, but in the presence of acetylated microtubules their ability to fluctuate in size is inhibited contributing to steatosis [161,163,165,167]. These results suggest targeted deacetylation of key regulatory proteins may support hepatic homeostasis through the interaction of mitochondrial, nuclear, and cytosolic processes to ameliorate ALD. Akin to NAFLD studies, resveratrol demonstrated a moderate level of protection [168]. Another group investigated the application of Vitexin to prevent alcohol-induced liver injury [160]. This study found that Vitexin decreased acetylated p53, increased SIRT1 expression, and decreased apoptosis to improve liver function. Another group utilized NR to mediate the effects of chronic alcohol consumption through an increase in SIRT1 activity by elevating NAD^+^ concentrations and reducing oxidative stress and mitochondrial biosynthesis [169]. In another study, NMN was utilized to increase hepatic NAD^+^ concentrations in a mouse model to support sirtuin activity. This study significantly increased NAD^+^ and prevented alcohol-induced increases in plasma ALT and AST, potentially through altered MAPK (mitogen-activated protein kinase) signaling [170]. Further evidence showed that MAPK signaling was inhibited once ethanol consumption was halted, decreasing protein abundance of p53 and other mitochondrial pro-apoptotic proteins [171]. Recent research suggests that targeting HDAC1 deacetylase activity via tributyrin can reduce hepatic lipid accumulation through altered CPT1a expression [172]. Thus, targeting histone deacetylase and sirtuin activity across subcellular compartments (nuclear/mitochondrial) may provide an innovative approach for therapeutic intervention in the initiation and progression of ALD. 

### 4.4. Hepatocellular Carcinoma

The etiology of hepatocellular carcinoma (HCC) is highly diverse and includes factors such as Hepatitis B and C, exposure to environmental toxicants, NAFLD, MetS, cigarette smoking, and ALD [173]. This heterogeneity makes treating HCC a significant challenge, leading to over 800,000 deaths worldwide each year [173]. The prognosis for HCC patients is poor (five-year survival rate less than 20%) and its diagnosis is rising in the United States [174,175,176]. The aggressive and proliferative nature of HCC supports an urgent need to further characterize cellular dysregulation occurring across the spectrum of cancer phenotypes.

Biochemical processes supporting the progression and metastasis of HCC are multi-factorial, yet recent evidence suggests mitochondrial metabolism and biogenesis are key factors in tumor growth and survival [177]. Mitochondrial ROS accumulation in cancer cells coupled with the hypoxic tumor microenvironment leads to alterations in glycolysis (cytoplasmic), TCA cycle (mitochondrial), and oxidative phosphorylation (mitochondrial), as well as changes in the redox ratios of NAD(P):NAD(P)H [178]. The mitochondrial dyshomeostasis observed in HCC suggests a focus on protein acetylation and sirtuin deacetylase activity may reveal unknown mechanistic roles for protein acylation in the induction and propagation of tumor cells [179]. The known impact of subcellular Ac-CoA concentrations on the initiation and progression of HCC were recently highlighted, showing upregulation of ATP-citrate synthase (ACLY) in HCC [180]. This indicated a shift in Ac-CoA from the mitochondria to the nucleus which was confirmed by a consequent decrease in mitochondrial protein acetylation and an increase in histone acetylation [180]. Thus, nuclear and mitochondrial concentrations of Ac-CoA provide a clear link between epigenetics and metabolism as a necessary regulatory feature in the development of HCC and is integrated alongside NAD^+^ and sirtuin activity.

**Figure 6:**
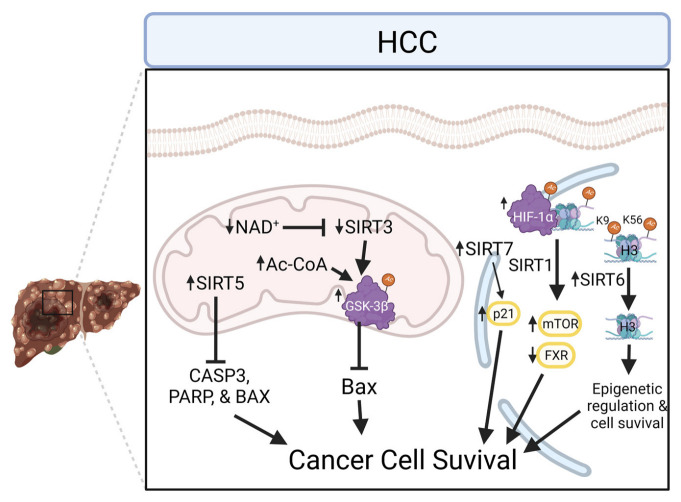
Pathological progression of hepatocellular carcinoma (HCC) via the manipulation of cellular defense mechanisms provides an unclear role for sirtuins. The decrease in NAD^+^ limits SIRT3 activity resulting in the acetylation of GSK-3β which regulates Bax inhibiting apoptosis. Furthermore, SIRT5 was observed to be upregulated to decrease apoptotic signals. Within the nucleus protein and histone acetylation influence transcription factors and epigenetic regulation of increase cell survival. Bax, BCL2-associated X protein; CASP3, caspase 3; H3, Histone H3; FXR, farnesoid X receptor; GSK-3β, glycogen synthase kinase-3β; mTOR, mammalian target of rapamycin; PPAR, peroxisome proliferator-activated receptor; SIRT, sirtuin.

Recent studies support mitochondrial sirtuins as prognostic markers or key regulators of HCC (Figure 6) [181]. Employing SIRT3 KO in a murine model of metabolic syndrome revealed that greater than 90% of SIRT3 KO mice developed HCC, providing an association with SIRT3 activity, metabolic syndrome, mitochondrial protein acetylation, and cancer progression [182]. SIRT3 also functions as a tumor suppressor in HCC through the PI3K/Akt pathway, where downregulation of SIRT3 and mitochondrial protein hyperacetylation increased cell proliferation and migration/invasion in HCC [183]. SIRT3 also deacetylates and activates glycogen synthase kinase-3β (GSK-3β), which induces the expression and mitochondrial translocation of the pro-apoptotic protein BCL2-associated X protein (Bax) [184]. Thus, SIRT3 may regulate mitochondrial-centered apoptosis through the SIRT3/GSK-3β/Bax signaling pathway. Sirtuins were found to be overexpressed in HCC cell lines compared to non-cancerous control cell lines. For example, increased SIRT5 abundance in HCC cells promoted cell proliferation and SIRT5 knockdown induces apoptosis in a mitochondrial-dependent manner [185]. Specifically, knockdown of SIRT5 by siRNA results in the upregulation of pro-apoptotic proteins caspase 3 (CASP3), PARP, and Bax while the apoptotic suppressor B-cell leukemia/lymphoma 2 (BCL-2) is downregulated, suggesting that the mitochondrial deacetylase activity of SIRT5 inhibits apoptosis in HCC cells [185]. 

Further demonstrating the coordinated nature of subcellular sirtuin activities, the non-mitochondrial sirtuins (SIRT1, 6, and 7) have been shown to play roles in HCC. Induction of SIRT6 activity in HCC cells is shown to decrease nuclear acetylation (specifically, H3K9 and H3K56), suggesting a potential role in epigenetic regulation and cell survival [186]. SIRT1 regulates HCC apoptotic pathways, as the inhibition of SIRT1 leads to hyperacetylation of hypoxia-inducible factor (HIF)-1α, inhibiting its function and impairing the hypoxic response of HCC cells thus inducing apoptosis [187,188]. SIRT1 also supports mitochondrial homeostasis given its role as a regulator of hepatic regenerative responses through deacetylation of farnesoid X receptor (FXR), histones, and mTOR signaling [189]. Given their role in cell growth and proliferation, nuclear and cytosolic sirtuin interaction with miRNAs has been examined extensively as a therapeutic target for HCC with implications for mitochondrial function [190]. The lesser studied SIRT7 was shown to have oncogenic potential in hepatocarcinogenesis whereby a regulatory loop was proposed with inhibition of transcriptional activation of p21(WAF1/Cip1) by SIRT7 through the repression of miR-125a-5p and miR-125b [191]. SIRT7 was found to be upregulated in HCC cells and a novel mechanism was identified supporting HCC resistance to therapy, as SIRT7 suppression increased doxorubicin-induced p53 activation through increased acetylation while inhibiting tumor growth and inducing apoptosis [192]. 

Therapeutic advances targeting sirtuins are currently being explored as studies suggest modulating SIRT3 abundance via Cyclin-dependent kinase (CDK) 4/6 inhibition may enhance HCC therapy when combined with sorafenib [193]. Furthermore, a mechanistic analysis revealed that SIRT3 downregulated both mRNA and protein levels of glutathione S-transferase pi 1 (GSTP1), a phase II detoxification enzyme. [194]. The authors found that this newly discovered SIRT3-GSTP1 interaction directed cellular apoptosis through c-Jun N-terminal kinase (JNK) activation. SIRT3 deacetylase activity and mTOR have also been indicated in metabolic dysfunction in HCC patients, revealing that SIRT3 and HIF-1α are prognostic indicators in early-stage HCC patients [195]. Moreover, SIRT3 overexpression also promotes ROS and apoptosis induced by the HCC therapy regorafenib, a multiple kinase inhibitor and mitochondrial toxicant, by accelerating mitochondrial depolarization induced by regorafenib and inducing mitochondrial dysfunction through impaired ETC function [196]. Aside from mitochondrially directed deacetylase activity, SIRT6 depletion was found to downregulate multidrug resistance protein 1 (MDR1) expression through the suppression of CCAAT/enhancer-binding protein (C/EBPβ), promoting enhanced chemosensitivity to HCC [197]. Overall, it remains unknown if sirtuin activation or inhibition is an appropriate therapeutic strategy for HCC, as targeting individual sirtuins for activation (SIRT3) and inhibition (SIRT6) may be indicated based on previous research [184,198,199].

### 4.5. Aging 

The population of aged individuals continues to rise globally and diseases associated with aging (e.g., cancer, Alzheimer’s, dementia) are placing a healthcare burden approaching $300 billion [200]. Indeed, other diseases, including metabolic syndrome, NAFLD, ALD, and T2D, can accelerate the aging process, resulting in increased mortality. Mitochondrial deterioration appears to be a driving factor in advanced aging and the decline of respiratory function, decreased mitochondrial oxidative capacity, and increased ROS [201,202,203]. Mitochondrial dysfunction and hypo-metabolism contribute to the accumulation of protein succinylation and acetylation over time resulting in an aged phenotype [204,205]. Aged mice (24 months) have significant accumulation of protein and histone acetylation compared to younger counterparts (3 month) [206]. ButyrylLys was found significantly increased on hepatic mitochondrial proteins in aged rodents [206]. This modification is not targeted by any mitochondrial sirtuin, but little is known about the impact of on protein function [207].

SIRT3 is decreased in aged hematopoietic stem cells (HSC) where SIRT3 deacetylase activity has been identified as a key regulator of mitochondrial homeostasis and tissue maintenance [208]. Here, the upregulation of SIRT3 improves HSC regenerative capacity directly through mitochondrial function [209]. Specifically, SIRT3 regulates the acylation of proteins involved in oxidative stress (SOD2), the ETC (complex I), and the TCA cycle (pyruvate dehydrogenase), emphasizing the importance of mitochondrial protein acetylation in aging related diseases and metabolic dysfunction [210]. Indeed, the utility of SIRT3 in maintaining HSC function plays a key role in the defense against aging-associated liver diseases, particularly in hepatic regeneration and repair processes [211]. Interestingly, overexpression of SIRT6 in male and female aged mice improved glucose output and homeostasis while providing significant improvements in mitochondrial function [212]. The mechanism elucidated by overexpressing SIRT6 showed increased hepatic gluconeogenic gene expression, de novo NAD^+^ synthesis, and increased glycerol release from adipose tissue in support of overall cellular health and mitochondrial function [212]. Furthermore, SIRT6 activated gene expression of proteins involved in beta-oxidation, TCA cycle, aerobic respiration, and amino acid catabolism while activating mitochondrial biogenesis and increased mitochondrial DNA content.

Decreased NAD^+^ stores influence aging through increases in protein acylation [213]. A decrease in NAD^+^, a key sirtuin co-factor, is observed in aged mice and results in enhanced metabolic disease [214]. This decrease of NAD^+^ is believed to be a driver of the biochemical phenotype of aging within humans, whereby glycolysis is impacted in aging individuals, resulting in decreased glycolytic output [213,215,216]. Additionally, different SIRT3 single nucleotide polymorphisms (SNPs) have been identified to alter SIRT3 activity [217]. An association was made between a synonymous SNP in SIRT3 and increased male survival and longevity. A nonsynonymous SNP in the catalytic domain of SIRT3 was also associated with metabolic syndrome in patients with NAFLD. This SNP reduced the catalytic activity of recombinant SIRT3 by 34%. Thus, reduced SIRT3 deacetylase activity may contribute to ageing and enhances metabolic disease in humans.

Circadian rhythm is another important biological aspect impacted by aging. Aging results in alterations in metabolic responses to light and dark cycles, such as NAD^+^ levels, glycolysis, and lipid metabolism [218]. In young mouse livers, a higher number of genes were found to be associated with protein acetylation compared to older livers, specifically among circadian genes related to NAD^+^ metabolic pathways [219]. Moreover, this effect was observed to be rescued using a model of caloric restriction in aged mice which improved age-dependent changes in protein acetylation but did not improve aging-related markers in humans (Table 1) [142,219]. Furthermore, the NAD^+^-dependent transcription factor, Period2 protein (PER2), is acetylated at Lys680 resulting in prolonged inhibition and accumulation of clock genes, leading to a disruption in circadian rhythms. Lys680 is regulated by SIRT1 and associated with decreased NAD^+^ due to aging, further supporting a role for sirtuins in age-related disruptions in metabolism and health. Interestingly, this study investigated the effects of NR, a NAD^+^ precursor, on aging and observed improved expression of genes required in mitochondrial respiration rhythms [220].

Metabolic health and aging are closely connected, and both are regulated through sirtuin activity. Resveratrol was used to activate SIRT1 and reduce the effects of aging by mimicking a fasted state [221]. Moreover, time-restricted feeding modulates sirtuin activity and NAD^+^ concentrations [135]. NAD^+^-boosting through NR and the targeted inhibition of PARP using olaparib improves mitochondrial function, activates the unfolded protein response, and increases lifespan [222]. In summary, these studies further support the potential therapeutic use of NR and NMN to increase NAD^+^ levels, thus activating sirtuins with the goal of reversing negative consequences of protein hyperacetylation on metabolism induced by the process of aging (Table 1) [141,223]. The manipulation of protein and histone acetylation remains an interesting target for improving metabolic health and hepatic function to counter the effects of aging.

## 5. Conclusions

The interplay between acyl-CoAs and protein modification is a critical regulator of mitochondrial health and the progression of hepatic pathologies. Given the contribution of liver disease to worldwide morbidity and mortality, further research is needed to understand how hepatic acetylation regulates mitochondrial homeostasis and overall hepatocellular function. Critical mechanistic aspects regarding proteomic and epigenetic regulation due to metabolic feedback are necessary for the development of effective therapeutics to support patients afflicted with the hepatic pathologies described above.

## Figures and Tables

**Table 1:** Pharmacological intervention of mitochondrial acyl post-translational modifications related to hepatic disease progression in patients. Abbreviations: AMPK, Adenosine monophosphate-activated protein kinase; d, day; DM, diabetes mellitus; FOXO, forkhead box1; MetS, metabolic syndrome; NAD^+^, nicotinamide adenine dinucleotide; NAFLD, non-alcoholic fatty liver disease; NF-κB, nuclear factor kappa b; NMN, nicotinamide mononucleotide; NR, Nicotinamide riboside; PPAR, peroxisome proliferator-activated receptor; SREBP1, sterol regulatory element binding protein 1; SIRT1, sirtuin 1.

Condition	Intervention	System	MOA	Outcomes	Ref.
MetS	Resveratrol	41 overweight men and women: double-blind clinical trial. 150 mg/d of resveratrol (*n* = 20) or placebo (*n* = 21) for 6 months (NCT02565979). 74 men with MetS: 3 groups. 1000 mg/d resveratrol (*n* = 21), 150 mg/d resveratrol (*n* = 21) and placebo (*n* = 24) for 16 weeks (NCT01412645).	SIRT1 agonist	No difference was observed in insulin sensitivity, body composition, blood pressure, energy metabolism, but there was a decrease in glycated hemoglobin. No improvement in inflammatory status, glucose homeostasis, plod pressure or hepatic lipid content.	[131,132]
	Time-restricted feeding	30 participants with metabolic syndrome. Restricted eating to 10 hours a day. 344 participants with type 2 diabetes: 4 groups. No change to meal timing or light exposure; early time-restricted feeding; light therapy; early time-restricted feeding and light therapy (NCT04155619).	Improve circadian rhythms, increase expression of AMPK, FOXO, sirtuins, and ketogenesis.	Publication pending.	[135,136]
	Metformin	50 patients: 3 groups. All treated with hypocaloric diet. Obese normoglycemic (*n* = 18); pre- diabetic treated with metformin (*n* = 16); pre-DM obese patients treated with metformin (*n* = 16).		Increased SIRT6 expression and decreased NF-κB, PPAR, SREBP1 expression in obese pre-diabetic patients treated with metformin.	[118]
	NR	40 male participants: 2 groups. 1 g nicotinamide riboside twice daily; placebo for 12 weeks (NCT02303483).	Increase NAD^+^	Failed to improve insulin sensitivity, whole-body glucose, body composition in obese and insulin- resistant men.	[137]
NAFLD	Resveratrol	Patients with NAFLD (*n* = 50): double blind clinical trial. 500 mg/d resveratrol (*n* = 25) or placebo (*n* = 25) for 12 weeks (NCT02030977).	SIRT1 agonist	Change in hepatic steatosis and inflammation	[102]
	Nicotinamide	61 participants: 2 groups. Both groups receiving anti-diabetic therapy (control, *n* = 30) with one supplemented with 1g/d nicotinamide (*n* = 31) for 12 weeks (NCT03850886).	Increase NAD^+^	Improved steatosis score, metabolic abnormalities and quality of life but failed to diminish liver fibrosis or steatosis.	[138]
	NMN	25 female participants: double-blind trail, 2 groups. 250 mg/d of NMN (*n* = 13) or placebo (*n* = 12) for 10 weeks (NCT03151239).	Increase NAD^+^	Improves skeletal muscle insulin signaling, insulin sensitivity and muscle remodeling in post-menopausal prediabetic women.	[139]
	Time-restricted Feeding	88 participants: 2 groups. Time restricted feeding (8 hour feeding window); Continuous energy restriction (NCT03786523).		Publication pending.	
Cancer	Time-restricted Eating	40 participants: single group assignment. Time-restricted eating (10 hour feeding window) for 14 days (NCT04243512).		Reduced fatigue.	[135,140]
Aging	NMN	66 participants: 2 groups. 300 mg/d NMN or placebo for 60 days (NCT04228640). 90 participants: 6 groups. 300 mg/d NMN (*n* = 20) or placebo (*n* = 7); 600 mg/d NMN (*n* = 20) or placebo (*n* = 7); 900 mg/d NMN (*n* = 10) or placebo (*n* = 6) for 60 days (NCT04823260).	Increase NAD^+^	Significantly elongated telomere length in lymphocytes, monocytes, and dendritic cells. Publication pending.	[141]
	Caloric Restriction	71 participants: 3 groups. Calorie restricting (*n* = 30); normal-eating controls (*n* = 16); obese comparison (*n* = 25) (NCT01256840).		Did not show delayed aging in either telomere length or T cell senescence markers.	[142]

## Abbreviations

AC, alcohol-associated cirrhosis; ACC, acetyl-CoA carboxylase; Ac-CoA, acetyl-CoA; ACS, Acyl-CoA synthase; Acox1, acyl-CoA oxidase 1; ADH, alcohol dehydrogenase; ALDH, aldehyde dehydrogenase; ALT, alanine aminotransferases; AhR, hydrocarbon receptor; AMP, adenosine monophosphate; AMPK, Adenosine monophosphate-activated protein kinase; ALD, Alcohol-associated liver disease; ASH, alcohol-associated steatohepatitis; AST, aspartate aminotransferases; ATP, Adenosine triphosphate; Bax, BCL2-associated X protein; BCL-2, B-cell leukemia/lymphoma 2; BMI, body mass index; CASP3, caspase 3; CCA, cholangiocarcinoma, C/EBPβ, CCAAT/enhancer-binding protein; CDK4, cyclin-dependent kinase 4; CDK6, cyclin-dependent kinase 6; CPT1a, carnitine palmitoyltransferase 1a; CYP2E1, cytochrome P450 family 2 subfamily E member 1; d, day; DNA, deoxyribonucleic acid; ESLD, end-stage liver disease; FAS, fatty acid synthase; Fox01, forkhead box1; FXR, farnesoid X receptor; F1P, fructose-1-kinase; GAPDH, glyceraldehyde 3-phosphate dehydrogenase; GCK, glucokinase; GCKR, glucokinase regulatory protein; GLUT2, Glucose transporter 2; GWAS, genome-wide association study; HCC, hepatocellular carcinoma; HDAC, histone deacetylases; HFD, high-fat diet; HGM-CoA, 3-hydroxy-3-methylglutaryl CoA; HIF-1α, hypoxia-inducible factor-1α; H3, Histone H3; IDH2, isocitrate dehydrogenase 2; KHK, ketohexokinase; LCAD, long-chain acyl-CoA dehydrogenase; LDLR, low density receptor gene; LKB1, liver kinase beta 1; Lys, lysine; MetS, metabolic syndrome; MDR1, multidrug resistance protein 1; mTOR, mammalian target of rapamycin; mTORC1, mTOR complex 1; mTORC1, mTOR complex 2; NADH, nicotinamide adenine dinucleotide; NADPH, nicotinamide adenine dinucleotide phosphate; NAFLD, non-alcoholic fatty liver disease; NAMPT, nicotinamide phophoribosyltransferase; NASH, non-alcoholic steatohepatitis, NR, nicotinamide riboside; Nrf2, nuclear factor (erythroid-derived 2)-like 2; NQO1, NAD(P)H: quinone oxidoreductase; PDC, Pyruvate dehydrogenase complex; PGC-1α, proliferator-activated receptors and γ coactivator 1α; PKM2, pyruvate kinase M2; PPAR-γ, peroxisome proliferator-activated receptor gamma; PP2A, serine/threonine protein phosphatase 2A; PTM, post-translational modification; RAPTOR, regulatory-associated protein of mammalian target rapamycin; SCD1, stearoyl-Coenzyme A desaturase-1; SREBP1, sterol regulatory element binding protein 1; SNP, single nucleotide polymorphisms; SIRT, sirtuin; STZ, streptozotocin; SOD2, superoxide dismutase; TCA, citric acid cycle; T2D, type 2 diabetes; Vldl, very low density lipoprotein.

## References

[1] Asrani S.K., Devarbhavi H., Eaton J., Kamath P.S. (2019). Burden of liver diseases in the world. J. Hepatol..

[2] Seto W.K., Mandell M.S. (2021). Chronic liver disease: Global perspectives and future challenges to delivering quality health care. PLoS ONE.

[3] Auger C., Alhasawi A., Contavadoo M., Appanna V.D. (2015). Dysfunctional mitochondrial bioenergetics and the pathogenesis of hepatic disorders. Front. Cell Dev. Biol..

[4] Ezhilarasan D. (2021). Mitochondria: A critical hub for hepatic stellate cells activation during chronic liver diseases. Hepatobiliary Pancreat. Dis. Int..

[5] Jennings E.Q., Fritz K.S., Galligan J.J. (2021). Biochemical genesis of enzymatic and non-enzymatic post-translational modifications. Mol. Asp. Med..

[6] Carrico C., Meyer J.G., He W., Gibson B.W., Verdin E. (2018). The Mitochondrial Acylome Emerges: Proteomics, Regulation by Sirtuins, and Metabolic and Disease Implications. Cell Metab..

[7] Meyer J.G., Softic S., Basisty N., Rardin M.J., Verdin E., Gibson B.W., Ilkayeva O., Newgard C.B., Kahn C.R., Schilling B. (2018). Temporal dynamics of liver mitochondrial protein acetylation and succinylation and metabolites due to high fat diet and/or excess glucose or fructose. PLoS ONE.

[8] Bak S., Leon I.R., Jensen O.N., Hojlund K. (2013). Tissue specific phosphorylation of mitochondrial proteins isolated from rat liver, heart muscle, and skeletal muscle. J. Proteome Res..

[9] Stram A.R., Payne R.M. (2016). Post-translational modifications in mitochondria: Protein signaling in the powerhouse. Cell Mol. Life Sci..

[10] Parodi-Rullan R.M., Chapa-Dubocq X.R., Javadov S. (2018). Acetylation of Mitochondrial Proteins in the Heart: The Role of SIRT3. Front. Physiol..

[11] Ben-Moshe S., Itzkovitz S. (2019). Spatial heterogeneity in the mammalian liver. Nat. Rev. Gastroenterol. Hepatol..

[12] Papanicolaou K.N., O’Rourke B., Foster D.B. (2014). Metabolism leaves its mark on the powerhouse: Recent progress in post-translational modifications of lysine in mitochondria. Front. Physiol..

[13] Beltrao P., Bork P., Krogan N.J., van Noort V. (2013). Evolution and functional cross-talk of protein post-translational modifications. Mol. Syst. Biol..

[14] Ducker G.S., Rabinowitz J.D. (2017). One-Carbon Metabolism in Health and Disease. Cell Metab..

[15] Li X., Egervari G., Wang Y., Berger S.L., Lu Z. (2018). Regulation of chromatin and gene expression by metabolic enzymes and metabolites. Nat. Rev. Mol. Cell Biol..

[16] Sousa F.L., Thiergart T., Landan G., Nelson-Sathi S., Pereira I.A., Allen J.F., Lane N., Martin W.F. (2013). Early bioenergetic evolution. Philos. Trans. R. Soc. Lond. B Biol. Sci..

[17] UniProt Consortium (2021). Controlled Vocabulary of Posttranslational Modifications (PTM). https://www.uniprot.org/docs/ptmlist.

[18] Wang Z.A., Cole P.A. (2020). The Chemical Biology of Reversible Lysine Post-translational Modifications. Cell Chem. Biol..

[19] Trefely S., Lovell C.D., Snyder N.W., Wellen K.E. (2020). Compartmentalised acyl-CoA metabolism and roles in chromatin regulation. Mol. Metab..

[20] Gaffney D.O., Jennings E.Q., Anderson C.C., Marentette J.O., Shi T., Schou Oxvig A.M., Streeter M.D., Johannsen M., Spiegel D.A., Chapman E. (2020). Non-enzymatic Lysine Lactoylation of Glycolytic Enzymes. Cell Chem. Biol..

[21] James A.M., Hoogewijs K., Logan A., Hall A.R., Ding S., Fearnley I.M., Murphy M.P. (2017). Non-enzymatic N-acetylation of Lysine Residues by AcetylCoA Often Occurs via a Proximal S-acetylated Thiol Intermediate Sensitive to Glyoxalase II. Cell Rep..

[22] Goodman R.P., Calvo S.E., Mootha V.K. (2018). Spatiotemporal compartmentalization of hepatic NADH and NADPH metabolism. J. Biol. Chem..

[23] Trefely S., Huber K., Liu J., Noji M., Stransky S., Singh J., Doan M.T., Lovell C.D., von Krusenstiern E., Jiang H. (2022). Quantitative subcellular acyl-CoA analysis reveals distinct nuclear metabolism and isoleucine-dependent histone propionylation. Mol. Cell.

[24] Bhatt D.P., Mills C.A., Anderson K.A., Henriques B.J., Lucas T.G., Francisco S., Liu J., Ilkayeva O.R., Adams A.E., Kulkarni S.R. (2022). Deglutarylation of glutaryl-CoA dehydrogenase by deacylating enzyme SIRT5 promotes lysine oxidation in mice. J. Biol. Chem..

[25] Harris P.S., Gomez J.D., Backos D.S., Fritz K.S. (2017). Characterizing Sirtuin 3 Deacetylase Affinity for Aldehyde Dehydrogenase 2. Chem. Res. Toxicol..

[26] Jennings E.Q., Ray J.D., Zerio C.J., Trujillo M.N., McDonald D.M., Chapman E., Spiegel D.A., Galligan J.J. (2021). Sirtuin 2 Regulates Protein LactoylLys Modifications. Chembiochem.

[27] Jin J., He B., Zhang X., Lin H., Wang Y. (2016). SIRT2 Reverses 4-Oxononanoyl Lysine Modification on Histones. J. Am. Chem. Soc.

[28] Du J., Zhou Y., Su X., Yu J.J., Khan S., Jiang H., Kim J., Woo J., Kim J.H., Choi B.H. (2011). Sirt5 is a NAD-dependent protein lysine demalonylase and desuccinylase. Science.

[29] Tan M., Peng C., Anderson K.A., Chhoy P., Xie Z., Dai L., Park J., Chen Y., Huang H., Zhang Y. (2014). Lysine glutarylation is a protein posttranslational modification regulated by SIRT5. Cell Metab..

[30] North B.J., Verdin E. (2004). Sirtuins: Sir2-related NAD-dependent protein deacetylases. Genome Biol..

[31] Kupis W., Palyga J., Tomal E., Niewiadomska E. (2016). The role of sirtuins in cellular homeostasis. J. Physiol. Biochem..

[32] Neubs H.P. (1991). The nursing shortage: Crisis as opportunity. Fla Nurse.

[33] Long D., Wu H., Tsang A.W., Poole L.B., Yoza B.K., Wang X., Vachharajani V., Furdui C.M., McCall C.E. (2017). The Oxidative State of Cysteine Thiol 144 Regulates the SIRT6 Glucose Homeostat. Sci. Rep..

[34] Michishita E., Park J.Y., Burneskis J.M., Barrett J.C., Horikawa I. (2005). Evolutionarily conserved and nonconserved cellular localizations and functions of human SIRT proteins. Mol. Biol. Cell.

[35] Garrity J., Gardner J.G., Hawse W., Wolberger C., Escalante-Semerena J.C. (2007). N-lysine propionylation controls the activity of propionyl-CoA synthetase. J. Biol. Chem..

[36] Tomaselli D., Steegborn C., Mai A., Rotili D. (2020). Sirt4: A Multifaceted Enzyme at the Crossroads of Mitochondrial Metabolism and Cancer. Front. Oncol..

[37] Abril Y.L.N., Fernandez I.R., Hong J.Y., Chiang Y.L., Kutateladze D.A., Zhao Q., Yang M., Hu J., Sadhukhan S., Li B. (2021). Pharmacological and genetic perturbation establish SIRT5 as a promising target in breast cancer. Oncogene.

[38] Zhang P., Tu B., Wang H., Cao Z., Tang M., Zhang C., Gu B., Li Z., Wang L., Yang Y. (2014). Tumor suppressor p53 cooperates with SIRT6 to regulate gluconeogenesis by promoting FoxO1 nuclear exclusion. Proc. Natl. Acad. Sci. USA.

[39] Shi M.Y., Bang I.H., Han C.Y., Lee D.H., Park B.H., Bae E.J. (2020). Statin suppresses sirtuin 6 through miR-495, increasing FoxO1-dependent hepatic gluconeogenesis. Theranostics.

[40] Rajabi N., Galleano I., Madsen A.S., Olsen C.A. (2018). Targeting Sirtuins: Substrate Specificity and Inhibitor Design. Prog. Mol. Biol. Transl. Sci..

[41] Zhang D., Tang Z., Huang H., Zhou G., Cui C., Weng Y., Liu W., Kim S., Lee S., Perez-Neut M. (2019). Metabolic regulation of gene expression by histone lactylation. Nature.

[42] Liu X., Wang H., Liang X., Roberts M.S. (2017). Hepatic Metabolism in Liver Health and Disease. Liver Pathophysiology.

[43] Lin H., Su X., He B. (2012). Protein lysine acylation and cysteine succination by intermediates of energy metabolism. ACS Chem. Biol..

[44] Adeva-Andany M.M., Perez-Felpete N., Fernandez-Fernandez C., Donapetry-Garcia C., Pazos-Garcia C. (2016). Liver glucose metabolism in humans. Biosci. Rep..

[45] Jones J.G. (2016). Hepatic glucose and lipid metabolism. Diabetologia.

[46] Yin S., Liu L., Gan W. (2021). The Roles of Post-Translational Modifications on mTOR Signaling. Int. J. Mol. Sci..

[47] Assifi M.M., Suchankova G., Constant S., Prentki M., Saha A.K., Ruderman N.B. (2005). AMP-activated protein kinase and coordination of hepatic fatty acid metabolism of starved/carbohydrate-refed rats. Am. J. Physiol. Endocrinol. Metab..

[48] Rodgers J.T., Puigserver P. (2007). Fasting-dependent glucose and lipid metabolic response through hepatic sirtuin 1. Proc. Natl. Acad. Sci. USA.

[49] Feige J.N., Lagouge M., Canto C., Strehle A., Houten S.M., Milne J.C., Lambert P.D., Mataki C., Elliott P.J., Auwerx J. (2008). Specific SIRT1 activation mimics low energy levels and protects against diet-induced metabolic disorders by enhancing fat oxidation. Cell Metab..

[50] Houtkooper R.H., Pirinen E., Auwerx J. (2012). Sirtuins as regulators of metabolism and healthspan. Nat. Rev. Mol. Cell Biol..

[51] He A., Chen X., Tan M., Chen Y., Lu D., Zhang X., Dean J.M., Razani B., Lodhi I.J. (2020). Acetyl-CoA Derived from Hepatic Peroxisomal beta-Oxidation Inhibits Autophagy and Promotes Steatosis via mTORC1 Activation. Mol. Cell.

[52] Rui L. (2014). Energy metabolism in the liver. Compr. Physiol..

[53] Ruderman N.B., Xu X.J., Nelson L., Cacicedo J.M., Saha A.K., Lan F., Ido Y. (2010). AMPK and SIRT1: A long-standing partnership?. Am. J. Physiol. Endocrinol. Metab..

[54] Canto C., Gerhart-Hines Z., Feige J.N., Lagouge M., Noriega L., Milne J.C., Elliott P.J., Puigserver P., Auwerx J. (2009). AMPK regulates energy expenditure by modulating NAD+ metabolism and SIRT1 activity. Nature.

[55] Tikhanovich I., Cox J., Weinman S.A. (2013). Forkhead box class O transcription factors in liver function and disease. J. Gastroenterol. Hepatol..

[56] Brunet A., Sweeney L.B., Sturgill J.F., Chua K.F., Greer P.L., Lin Y., Tran H., Ross S.E., Mostoslavsky R., Cohen H.Y. (2004). Stress-dependent regulation of FOXO transcription factors by the SIRT1 deacetylase. Science.

[57] Webster B.R., Scott I., Traba J., Han K., Sack M.N. (2014). Regulation of autophagy and mitophagy by nutrient availability and acetylation. Biochim. Biophys. Acta..

[58] Viollet B., Foretz M., Guigas B., Horman S., Dentin R., Bertrand L., Hue L., Andreelli F. (2006). Activation of AMP-activated protein kinase in the liver: A new strategy for the management of metabolic hepatic disorders. J. Physiol..

[59] Czaja M.J., Ding W.X., Donohue T.M., Friedman S.L., Kim J.S., Komatsu M., Lemasters J.J., Lemoine A., Lin J.D., Ou J.H. (2013). Functions of autophagy in normal and diseased liver. Autophagy.

[60] Youle R.J., van der Bliek A.M. (2012). Mitochondrial fission, fusion, and stress. Science.

[61] Son S.M., Park S.J., Lee H., Siddiqi F., Lee J.E., Menzies F.M., Rubinsztein D.C. (2019). Leucine Signals to mTORC1 via Its Metabolite Acetyl-Coenzyme A. Cell Metab..

[62] Park S.H., Ozden O., Liu G., Song H.Y., Zhu Y., Yan Y., Zou X., Kang H.J., Jiang H., Principe D.R. (2016). SIRT2-Mediated Deacetylation and Tetramerization of Pyruvate Kinase Directs Glycolysis and Tumor Growth. Cancer Res..

[63] Lv L., Xu Y.P., Zhao D., Li F.L., Wang W., Sasaki N., Jiang Y., Zhou X., Li T.T., Guan K.L. (2013). Mitogenic and oncogenic stimulation of K433 acetylation promotes PKM2 protein kinase activity and nuclear localization. Mol. Cell.

[64] Cotter T.G., Rinella M. (2020). Nonalcoholic Fatty Liver Disease 2020: The State of the Disease. Gastroenterology.

[65] Wong R.J., Aguilar M., Cheung R., Perumpail R.B., Harrison S.A., Younossi Z.M., Ahmed A. (2015). Nonalcoholic steatohepatitis is the second leading etiology of liver disease among adults awaiting liver transplantation in the United States. Gastroenterology.

[66] Schwimmer J.B., Deutsch R., Kahen T., Lavine J.E., Stanley C., Behling C. (2006). Prevalence of fatty liver in children and adolescents. Pediatrics.

[67] Benedict M., Zhang X. (2017). Non-alcoholic fatty liver disease: An expanded review. World J. Hepatol..

[68] Khan R.S., Bril F., Cusi K., Newsome P.N. (2019). Modulation of Insulin Resistance in Nonalcoholic Fatty Liver Disease. Hepatology.

[69] Softic S., Cohen D.E., Kahn C.R. (2016). Role of Dietary Fructose and Hepatic De Novo Lipogenesis in Fatty Liver Disease. Dig. Dis. Sci..

[70] Farzanegi P., Dana A., Ebrahimpoor Z., Asadi M., Azarbayjani M.A. (2019). Mechanisms of beneficial effects of exercise training on non-alcoholic fatty liver disease (NAFLD): Roles of oxidative stress and inflammation. Eur. J. Sport Sci..

[71] Kendrick A.A., Choudhury M., Rahman S.M., McCurdy C.E., Friederich M., Van Hove J.L., Watson P.A., Birdsey N., Bao J., Gius D. (2011). Fatty liver is associated with reduced SIRT3 activity and mitochondrial protein hyperacetylation. Biochem. J..

[72] Bao J., Scott I., Lu Z., Pang L., Dimond C.C., Gius D., Sack M.N. (2010). SIRT3 is regulated by nutrient excess and modulates hepatic susceptibility to lipotoxicity. Free Radic. Biol. Med..

[73] Bharathi S.S., Zhang Y., Mohsen A.W., Uppala R., Balasubramani M., Schreiber E., Uechi G., Beck M.E., Rardin M.J., Vockley J. (2013). Sirtuin 3 (SIRT3) protein regulates long-chain acyl-CoA dehydrogenase by deacetylating conserved lysines near the active site. J. Biol. Chem..

[74] Zhang D., Liu Z.X., Choi C.S., Tian L., Kibbey R., Dong J., Cline G.W., Wood P.A., Shulman G.I. (2007). Mitochondrial dysfunction due to long-chain Acyl-CoA dehydrogenase deficiency causes hepatic steatosis and hepatic insulin resistance. Proc. Natl. Acad. Sci. USA.

[75] Kurtz D.M., Rinaldo P., Rhead W.J., Tian L., Millington D.S., Vockley J., Hamm D.A., Brix A.E., Lindsey J.R., Pinkert C.A. (1998). Targeted disruption of mouse long-chain acyl-CoA dehydrogenase gene reveals crucial roles for fatty acid oxidation. Proc. Natl. Acad. Sci. USA.

[76] Hirschey M.D., Shimazu T., Goetzman E., Jing E., Schwer B., Lombard D.B., Grueter C.A., Harris C., Biddinger S., Ilkayeva O.R. (2010). SIRT3 regulates mitochondrial fatty-acid oxidation by reversible enzyme deacetylation. Nature.

[77] Barroso E., Rodriguez-Rodriguez R., Zarei M., Pizarro-Degado J., Planavila A., Palomer X., Villarroya F., Vazquez-Carrera M. (2020). SIRT3 deficiency exacerbates fatty liver by attenuating the HIF1alpha-LIPIN 1 pathway and increasing CD36 through Nrf2. Cell Commun. Signal.

[78] Osborne B., Reznick J., Wright L.E., Sinclair D.A.C., Gregory J. (2022). Turner, Nigel Liver-specific overexpression of SIRT3 enhances oxidative metabolism, but does not impact metabolic defects induced by high fat feeding in mice. Biochem. Biophys. Res. Commun..

[79] Schwer B., Bunkenborg J., Verdin R.O., Andersen J.S., Verdin E. (2006). Reversible lysine acetylation controls the activity of the mitochondrial enzyme acetyl-CoA synthetase 2. Proc. Natl. Acad. Sci. USA.

[80] Hirschey M.D., Shimazu T., Capra J.A., Pollard K.S., Verdin E. (2011). SIRT1 and SIRT3 deacetylate homologous substrates: AceCS1,2 and HMGCS1,2. Aging.

[81] Arroyave-Ospina J.C., Wu Z., Geng Y., Moshage H. (2021). Role of Oxidative Stress in the Pathogenesis of Non-Alcoholic Fatty Liver Disease: Implications for Prevention and Therapy. Antioxid..

[82] Qiu X., Brown K., Hirschey M.D., Verdin E., Chen D. (2010). Calorie restriction reduces oxidative stress by SIRT3-mediated SOD2 activation. Cell Metab..

[83] Tao R., Coleman M.C., Pennington J.D., Ozden O., Park S.H., Jiang H., Kim H.S., Flynn C.R., Hill S., Hayes McDonald W. (2010). Sirt3-mediated deacetylation of evolutionarily conserved lysine 122 regulates MnSOD activity in response to stress. Mol. Cell.

[84] He J., Hu B., Shi X., Weidert E.R., Lu P., Xu M., Huang M., Kelley E.E., Xie W. (2013). Activation of the aryl hydrocarbon receptor sensitizes mice to nonalcoholic steatohepatitis by deactivating mitochondrial sirtuin deacetylase Sirt3. Mol. Cell Biol..

[85] He A., Dean J.M., Lu D., Chen Y., Lodhi I.J. (2020). Hepatic peroxisomal beta-oxidation suppresses lipophagy via RPTOR acetylation and MTOR activation. Autophagy.

[86] Bertolio R., Napoletano F., Mano M., Maurer-Stroh S., Fantuz M., Zannini A., Bicciato S., Sorrentino G., Del Sal G. (2019). Sterol regulatory element binding protein 1 couples mechanical cues and lipid metabolism. Nat. Commun..

[87] Chen X.Y., Cai C.Z., Yu M.L., Feng Z.M., Zhang Y.W., Liu P.H., Zeng H., Yu C.H. (2019). LB100 ameliorates nonalcoholic fatty liver disease via the AMPK/Sirt1 pathway. World J. Gastroenterol..

[88] Nassir F. (2020). Role of acetylation in nonalcoholic fatty liver disease: A focus on SIRT1 and SIRT3. Explor. Med..

[89] Kim H., Mendez R., Chen X., Fang D., Zhang K. (2015). Lysine Acetylation of CREBH Regulates Fasting-Induced Hepatic Lipid Metabolism. Mol. Cell Biol..

[90] Bricambert J., Miranda J., Benhamed F., Girard J., Postic C., Dentin R. (2010). Salt-inducible kinase 2 links transcriptional coactivator p300 phosphorylation to the prevention of ChREBP-dependent hepatic steatosis in mice. J. Clin. Invest..

[91] Chen S., Feng B., George B., Chakrabarti R., Chen M., Chakrabarti S. (2010). Transcriptional coactivator p300 regulates glucose-induced gene expression in endothelial cells. Am. J. Physiol. Endocrinol. Metab..

[92] Dall M., Hassing A.S., Treebak J.T. (2021). NAD(+) and NAFLD caution, causality and careful optimism. J. Physiol..

[93] Zhou C.C., Yang X., Hua X., Liu J., Fan M.B., Li G.Q., Song J., Xu T.Y., Li Z.Y., Guan Y.F. (2016). Hepatic NAD(+) deficiency as a therapeutic target for non-alcoholic fatty liver disease in ageing. Br. J. Pharm..

[94] Dahl T.B., Haukeland J.W., Yndestad A., Ranheim T., Gladhaug I.P., Damas J.K., Haaland T., Loberg E.M., Arntsen B., Birkeland K. (2010). Intracellular nicotinamide phosphoribosyltransferase protects against hepatocyte apoptosis and is down-regulated in nonalcoholic fatty liver disease. J. Clin. Endocrinol. Metab..

[95] Dall M., Hassing A.S., Niu L., Nielsen T.S., Ingerslev L.R., Sulek K., Trammell S.A.J., Gillum M.P., Barres R., Larsen S. (2021). Hepatocyte-specific perturbation of NAD(+) biosynthetic pathways in mice induces reversible nonalcoholic steatohepatitis-like phenotypes. J. Biol. Chem..

[96] Wang L.F., Wang X.N., Huang C.C., Hu L., Xiao Y.F., Guan X.H., Qian Y.S., Deng K.Y., Xin H.B. (2017). Inhibition of NAMPT aggravates high fat diet-induced hepatic steatosis in mice through regulating Sirt1/AMPKalpha/SREBP1 signaling pathway. Lipids Health Dis..

[97] Kozako T., Suzuki T., Yoshimitsu M., Arima N., Honda S., Soeda S. (2014). Anticancer agents targeted to sirtuins. Molecules.

[98] Zhao E., Hou J., Ke X., Abbas M.N., Kausar S., Zhang L., Cui H. (2019). The Roles of Sirtuin Family Proteins in Cancer Progression. Cancers.

[99] De Lorenzo S., Tovoli F., Mazzotta A., Vasuri F., Edeline J., Malvi D., Boudjema K., Renzulli M., Jeddou H., D’Errico A. (2020). Non-Alcoholic Steatohepatitis as a Risk Factor for Intrahepatic Cholangiocarcinoma and Its Prognostic Role. Cancers.

[100] Pant K., Peixoto E., Richard S., Gradilone S.A. (2020). Role of Histone Deacetylases in Carcinogenesis: Potential Role in Cholangiocarcinoma. Cells.

[101] Asgary S., Karimi R., Momtaz S., Naseri R., Farzaei M.H. (2019). Effect of resveratrol on metabolic syndrome components: A systematic review and meta-analysis. Rev. Endocr. Metab. Disord..

[102] Chen S., Zhao X., Ran L., Wan J., Wang X., Qin Y., Shu F., Gao Y., Yuan L., Zhang Q. (2015). Resveratrol improves insulin resistance, glucose and lipid metabolism in patients with non-alcoholic fatty liver disease: A randomized controlled trial. Dig. Liver Dis..

[103] Karimi M., Abiri B., Guest P.C., Vafa M. (2022). Therapeutic Effects of Resveratrol on Nonalcoholic Fatty Liver Disease Through Inflammatory, Oxidative Stress, Metabolic, and Epigenetic Modifications. Methods Mol. Biol..

[104] Wei S., Yu X. (2021). Efficacy of resveratrol supplementation on liver enzymes in patients with non-alcoholic fatty liver disease: A systematic review and meta-analysis. Complement. Ther. Med..

[105] Rafiee S., Mohammadi H., Ghavami A., Sadeghi E., Safari Z., Askari G. (2021). Efficacy of resveratrol supplementation in patients with nonalcoholic fatty liver disease: A systematic review and meta-analysis of clinical trials. Complement Clin. Pract..

[106] Seo D.B., Jeong H.W., Lee S.J., Lee S.J. (2014). Coumestrol induces mitochondrial biogenesis by activating Sirt1 in cultured skeletal muscle cells. J. Agric. Food Chem..

[107] Teodoro J.S., Duarte F.V., Gomes A.P., Varela A.T., Peixoto F.M., Rolo A.P., Palmeira C.M. (2013). Berberine reverts hepatic mitochondrial dysfunction in high-fat fed rats: A possible role for SirT3 activation. Mitochondrion.

[108] Xu X., Zhu X.P., Bai J.Y., Xia P., Li Y., Lu Y., Li X.Y., Gao X. (2019). Berberine alleviates nonalcoholic fatty liver induced by a high-fat diet in mice by activating SIRT3. FASEB J..

[109] Zeng X., Yang J., Hu O., Huang J., Ran L., Chen M., Zhang Y., Zhou X., Zhu J., Zhang Q. (2019). Dihydromyricetin Ameliorates Nonalcoholic Fatty Liver Disease by Improving Mitochondrial Respiratory Capacity and Redox Homeostasis Through Modulation of SIRT3 Signaling. Antioxid. Redox Signal.

[110] Huang L., Zeng X., Li B., Wang C., Zhou M., Lang H., Yi L., Mi M. (2021). Dihydromyricetin attenuates palmitic acid-induced oxidative stress by promoting autophagy via SIRT3-ATG4B signaling in hepatocytes. Nutr. Metab..

[111] Ali A., Zhang Y., Fu M., Pei Y., Wu L., Wang R., Yang G. (2020). Cystathionine gamma-lyase/H2S system suppresses hepatic acetyl-CoA accumulation and nonalcoholic fatty liver disease in mice. Life Sci..

[112] Alberti K.G., Eckel R.H., Grundy S.M., Zimmet P.Z., Cleeman J.I., Donato K.A., Fruchart J.C., James W.P., Loria C.M., Smith S.C. (2009). Harmonizing the metabolic syndrome: A joint interim statement of the International Diabetes Federation Task Force on Epidemiology and Prevention; National Heart, Lung, and Blood Institute; American Heart Association; World Heart Federation; International Atherosclerosis Society; and International Association for the Study of Obesity. Circulation.

[113] Saklayen M.G. (2018). The Global Epidemic of the Metabolic Syndrome. Curr. Hypertens. Rep..

[114] Gallagher E.J., Leroith D., Karnieli E. (2011). The metabolic syndrome—From insulin resistance to obesity and diabetes. Med. Clin. N. Am..

[115] Moore J.X., Chaudhary N., Akinyemiju T. (2017). Metabolic Syndrome Prevalence by Race/Ethnicity and Sex in the United States, National Health and Nutrition Examination Survey, 1988-2012. Prev. Chronic Dis..

[116] Bureau U.S.C. Population Estimates by Age (18+): July 1, 2019. https://www.census.gov/data/tables/time-series/demo/popest/2010s-national-detail.html.

[117] Moschen A.R., Wieser V., Gerner R.R., Bichler A., Enrich B., Moser P., Ebenbichler C.F., Kaser S., Tilg H. (2013). Adipose tissue and liver expression of SIRT1, 3, and 6 increase after extensive weight loss in morbid obesity. J. Hepatol..

[118] D’Onofrio N., Pieretti G., Ciccarelli F., Gambardella A., Passariello N., Rizzo M.R., Barbieri M., Marfella R., Nicoletti G., Balestrieri M.L. (2019). Abdominal Fat SIRT6 Expression and Its Relationship with Inflammatory and Metabolic Pathways in Pre-Diabetic Overweight Patients. Int. J. Mol. Sci..

[119] Elhanati S., Kanfi Y., Varvak A., Roichman A., Carmel-Gross I., Barth S., Gibor G., Cohen H.Y. (2013). Multiple regulatory layers of SREBP1/2 by SIRT6. Cell Rep..

[120] Fuchs T., Loureiro M.P., Macedo L.E., Nocca D., Nedelcu M., Costa-Casagrande T.A. (2018). Animal models in metabolic syndrome. Rev. Col. Bras. Cir..

[121] Carreira M.C., Izquierdo A.G., Amil M., Casanueva F.F., Crujeiras A.B. (2018). Oxidative Stress Induced by Excess of Adiposity Is Related to a Downregulation of Hepatic SIRT6 Expression in Obese Individuals. Oxid. Med. Cell. Longev..

[122] Hirschey M.D., Shimazu T., Jing E., Grueter C.A., Collins A.M., Aouizerat B., Stancakova A., Goetzman E., Lam M.M., Schwer B. (2011). SIRT3 deficiency and mitochondrial protein hyperacetylation accelerate the development of the metabolic syndrome. Mol. Cell.

[123] Botezelli J.D., Cambri L.T., Ghezzi A.C., Dalia R.A., Voltarelli F.A., de Mello M.A. (2012). Fructose-rich diet leads to reduced aerobic capacity and to liver injury in rats. Lipids Health Dis..

[124] Sato N. (2007). Central role of mitochondria in metabolic regulation of liver pathophysiology. J. Gastroenterol. Hepatol..

[125] Kamata K., Mitsuya M., Nishimura T., Eiki J., Nagata Y. (2004). Structural basis for allosteric regulation of the monomeric allosteric enzyme human glucokinase. Structure.

[126] Herman M.A., Birnbaum M.J. (2021). Molecular aspects of fructose metabolism and metabolic disease. Cell Metab..

[127] Chyau C.C., Wang H.F., Zhang W.J., Chen C.C., Huang S.H., Chang C.C., Peng R.Y. (2020). Antrodan Alleviates High-Fat and High-Fructose Diet-Induced Fatty Liver Disease in C57BL/6 Mice Model via AMPK/Sirt1/SREBP-1c/PPARgamma Pathway. Int. J. Mol. Sci..

[128] Softic S., Meyer J.G., Wang G.X., Gupta M.K., Batista T.M., Lauritzen H., Fujisaka S., Serra D., Herrero L., Willoughby J. (2019). Dietary Sugars Alter Hepatic Fatty Acid Oxidation via Transcriptional and Post-translational Modifications of Mitochondrial Proteins. Cell Metab..

[129] Pfluger P.T., Herranz D., Velasco-Miguel S., Serrano M., Tschop M.H. (2008). Sirt1 protects against high-fat diet-induced metabolic damage. Proc. Natl. Acad. Sci. USA.

[130] Rodgers J.T., Lerin C., Haas W., Gygi S.P., Spiegelman B.M., Puigserver P. (2005). Nutrient control of glucose homeostasis through a complex of PGC-1alpha and SIRT1. Nature.

[131] de Ligt M., Bergman M., Fuentes R.M., Essers H., Moonen-Kornips E., Havekes B., Schrauwen-Hinderling V.B., Schrauwen P. (2020). No effect of resveratrol supplementation after 6 months on insulin sensitivity in overweight adults: A randomized trial. Am. J. Clin. Nutr..

[132] Kjaer T.N., Ornstrup M.J., Poulsen M.M., Stodkilde-Jorgensen H., Jessen N., Jorgensen J.O.L., Richelsen B., Pedersen S.B. (2017). No Beneficial Effects of Resveratrol on the Metabolic Syndrome: A Randomized Placebo-Controlled Clinical Trial. J. Clin. Endocrinol. Metab..

[133] Goyal S.N., Reddy N.M., Patil K.R., Nakhate K.T., Ojha S., Patil C.R., Agrawal Y.O. (2016). Challenges and issues with streptozotocin-induced diabetes A clinically relevant animal model to understand the diabetes pathogenesis and evaluate therapeutics. Chem. Biol. Interact..

[134] Trammell S.A., Weidemann B.J., Chadda A., Yorek M.S., Holmes A., Coppey L.J., Obrosov A., Kardon R.H., Yorek M.A., Brenner C. (2016). Nicotinamide Riboside Opposes Type 2 Diabetes and Neuropathy in Mice. Sci. Rep..

[135] Manoogian E.N., Chow L.S., Taub P.R., Laferrere B., Panda S. (2021). Time-restricted eating for the prevention and management of metabolic diseases. Endocr. Rev..

[136] Swiatkiewicz I., Mila-Kierzenkowska C., Wozniak A., Szewczyk-Golec K., Nuszkiewicz J., Wroblewska J., Rajewski P., Eussen S., Faerch K., Manoogian E.N.C. (2021). Pilot Clinical Trial of Time-Restricted Eating in Patients with Metabolic Syndrome. Nutrients.

[137] Dollerup O.L., Christensen B., Svart M., Schmidt M.S., Sulek K., Ringgaard S., Stodkilde-Jorgensen H., Moller N., Brenner C., Treebak J.T. (2018). A randomized placebo-controlled clinical trial of nicotinamide riboside in obese men: Safety, insulin-sensitivity, and lipid-mobilizing effects. Am. J. Clin. Nutr..

[138] El-Kady R.R., Ali A.K., El Wakeel L.M., Sabri N.A., Shawki M.A. (2022). Nicotinamide supplementation in diabetic nonalcoholic fatty liver disease patients: Randomized controlled trial. Adv. Chronic. Dis..

[139] Yoshino M., Yoshino J., Kayser B.D., Patti G.J., Franczyk M.P., Mills K.F., Sindelar M., Pietka T., Patterson B.W., Imai S.I. (2021). Nicotinamide mononucleotide increases muscle insulin sensitivity in prediabetic women. Science.

[140] Kleckner A., Reschke J.E., Altman B.J., Belcher E., Dunne R.F., Fleming F.J., Gilmore N., Jensen-Battaglia M., Kleckner I., Lin P.-J. (2021). A 10-hour time-restricted eating intervention to address cancer-related fatigue among cancer survivors. J. Clin. Oncol..

[141] Niu K.M., Bao T., Gao L., Ru M., Li Y., Jiang L., Ye C., Wang S., Wu X. (2021). The Impacts of Short-Term NMN Supplementation on Serum Metabolism, Fecal Microbiota, and Telomere Length in Pre-Aging Phase. Front. Nutr..

[142] Tomiyama A.J., Milush J.M., Lin J., Flynn J.M., Kapahi P., Verdin E., Sinclair E., Melov S., Epel E.S. (2017). Long-term calorie restriction in humans is not associated with indices of delayed immunologic aging: A descriptive study. Nutr. Healthy Aging.

[143] Seitz H.K., Bataller R., Cortez-Pinto H., Gao B., Gual A., Lackner C., Mathurin P., Mueller S., Szabo G., Tsukamoto H. (2018). Alcoholic liver disease. Nat. Rev. Dis. Primers.

[144] Zakhari S. (2006). Overview: How is alcohol metabolized by the body?. Alcohol. Res. Health.

[145] Wilson D.F., Matschinsky F.M. (2020). Ethanol metabolism: The good, the bad, and the ugly. Med. Hypotheses.

[146] Tuma D.J., Casey C.A. (2003). Dangerous byproducts of alcohol breakdown—Focus on adducts. Alcohol. Res. Health.

[147] Fritz K.S., Galligan J.J., Hirschey M.D., Verdin E., Petersen D.R. (2012). Mitochondrial acetylome analysis in a mouse model of alcohol-induced liver injury utilizing SIRT3 knockout mice. J. Proteome Res..

[148] Shepard B.D., Tuma D.J., Tuma P.L. (2010). Chronic ethanol consumption induces global hepatic protein hyperacetylation. Alcohol. Clin. Exp. Res..

[149] Lieber C.S., Leo M.A., Wang X., Decarli L.M. (2008). Alcohol alters hepatic FoxO1, p53, and mitochondrial SIRT5 deacetylation function. Biochem. Biophys. Res. Commun..

[150] Picklo M.J. (2008). Ethanol intoxication increases hepatic N-lysyl protein acetylation. Biochem. Biophys. Res. Commun..

[151] Marchner H., Tottmar O. (1978). A comparative study on the effects of disulfiram, cyanamide and 1-aminocyclopropanol on the acetaldehyde metabolism in rats. Acta. Pharm. Toxicol..

[152] Doody E.E., Groebner J.L., Walker J.R., Frizol B.M., Tuma D.J., Fernandez D.J., Tuma P.L. (2017). Ethanol metabolism by alcohol dehydrogenase or cytochrome P450 2E1 differentially impairs hepatic protein trafficking and growth hormone signaling. Am. J. Physiol. Gastrointest. Liver Physiol..

[153] Bertola A., Mathews S., Ki S.H., Wang H., Gao B. (2013). Mouse model of chronic and binge ethanol feeding (the NIAAA model). Nat. Protoc..

[154] Lieber C.S., DeCarli L.M. (1982). The feeding of alcohol in liquid diets: Two decades of applications and 1982 update. Alcohol. Clin. Exp. Res..

[155] Ghosh Dastidar S., Warner J.B., Warner D.R., McClain C.J., Kirpich I.A. (2018). Rodent Models of Alcoholic Liver Disease: Role of Binge Ethanol Administration. Biomolecules.

[156] Shepard B.D., Tuma P.L. (2009). Alcohol-induced protein hyperacetylation: Mechanisms and consequences. World J. Gastroenterol..

[157] Fritz K.S., Green M.F., Petersen D.R., Hirschey M.D. (2013). Ethanol metabolism modifies hepatic protein acylation in mice. PLoS ONE.

[158] Assiri M.A., Roy S.R., Harris P.S., Ali H., Liang Y., Shearn C.T., Orlicky D.J., Roede J.R., Hirschey M.D., Backos D.S. (2017). Chronic Ethanol Metabolism Inhibits Hepatic Mitochondrial Superoxide Dismutase via Lysine Acetylation. Alcohol. Clin. Exp. Res..

[159] Lee J.T., Gu W. (2013). SIRT1: Regulator of p53 Deacetylation. Genes Cancer.

[160] Yuan H., Duan S., Guan T., Yuan X., Lin J., Hou S., Lai X., Huang S., Du X., Chen S. (2020). Vitexin protects against ethanol-induced liver injury through Sirt1/p53 signaling pathway. Eur. J. Pharm..

[161] Hu M., Wang F., Li X., Rogers C.Q., Liang X., Finck B.N., Mitra M.S., Zhang R., Mitchell D.A., You M. (2012). Regulation of hepatic lipin-1 by ethanol: Role of AMP-activated protein kinase/sterol regulatory element-binding protein 1 signaling in mice. Hepatology.

[162] Ren R., Wang Z., Wu M., Wang H. (2020). Emerging Roles of SIRT1 in Alcoholic Liver Disease. Int. J. Biol. Sci..

[163] You M., Jogasuria A., Taylor C., Wu J. (2015). Sirtuin 1 signaling and alcoholic fatty liver disease. Hepatobiliary Surg. Nutr..

[164] Pandey S.C., Bohnsack J.P. (2020). Alcohol Makes Its Epigenetic Marks. Cell Metab..

[165] Kim J.S., Shukla S.D. (2006). Acute in vivo effect of ethanol (binge drinking) on histone H3 modifications in rat tissues. Alcohology.

[166] Joseph R.A., Shepard B.D., Kannarkat G.T., Rutledge T.M., Tuma D.J., Tuma P.L. (2008). Microtubule acetylation and stability may explain alcohol-induced alterations in hepatic protein trafficking. Hepatology.

[167] Shukla S.D., Aroor A.R., Restrepo R., Kharbanda K.K., Ibdah J.A. (2015). In Vivo Acute on Chronic Ethanol Effects in Liver: A Mouse Model Exhibiting Exacerbated Injury, Altered Metabolic and Epigenetic Responses. Biomolecules.

[168] Ajmo J.M., Liang X., Rogers C.Q., Pennock B., You M. (2008). Resveratrol alleviates alcoholic fatty liver in mice. Am. J. Physiol. Gastrointest. Liver Physiol..

[169] Wang S., Wan T., Ye M., Qiu Y., Pei L., Jiang R., Pang N., Huang Y., Liang B., Ling W. (2018). Nicotinamide riboside attenuates alcohol induced liver injuries via activation of SirT1/PGC-1alpha/mitochondrial biosynthesis pathway. Redox Biol..

[170] Assiri M.A., Ali H.R., Marentette J.O., Yun Y., Liu J., Hirschey M.D., Saba L.M., Harris P.S., Fritz K.S. (2019). Investigating RNA expression profiles altered by nicotinamide mononucleotide therapy in a chronic model of alcoholic liver disease. Hum. Genom..

[171] Pi A., Jiang K., Ding Q., Lai S., Yang W., Zhu J., Guo R., Fan Y., Chi L., Li S. (2021). Alcohol Abstinence Rescues Hepatic Steatosis and Liver Injury via Improving Metabolic Reprogramming in Chronic Alcohol-Fed Mice. Front. Pharm..

[172] Donde H., Ghare S., Joshi-Barve S., Zhang J., Vadhanam M.V., Gobejishvili L., Lorkiewicz P., Srivastava S., McClain C.J., Barve S. (2020). Tributyrin Inhibits Ethanol-Induced Epigenetic Repression of CPT-1A and Attenuates Hepatic Steatosis and Injury. Cell Mol. Gastroenterol. Hepatol..

[173] Barcena-Varela M., Lujambio A. (2021). The Endless Sources of Hepatocellular Carcinoma Heterogeneity. Cancers.

[174] Li Y., Xu S., Li J., Zheng L., Feng M., Wang X., Han K., Pi H., Li M., Huang X. (2016). SIRT1 facilitates hepatocellular carcinoma metastasis by promoting PGC-1alpha-mediated mitochondrial biogenesis. Oncotarget.

[175] Siegel R.L., Miller K.D., Jemal A. (2020). Cancer statistics, 2020. CA Cancer J. Clin..

[176] Tohme S., Yazdani H.O., Liu Y., Loughran P., van der Windt D.J., Huang H., Simmons R.L., Shiva S., Tai S., Tsung A. (2017). Hypoxia mediates mitochondrial biogenesis in hepatocellular carcinoma to promote tumor growth through HMGB1 and TLR9 interaction. Hepatology.

[177] Lee H.Y., Nga H.T., Tian J., Yi H.S. (2021). Mitochondrial Metabolic Signatures in Hepatocellular Carcinoma. Cells.

[178] Choudhury F.K. (2021). Mitochondrial Redox Metabolism: The Epicenter of Metabolism during Cancer Progression. Antioxidants.

[179] De Matteis S., Granato A.M., Napolitano R., Molinari C., Valgiusti M., Santini D., Foschi F.G., Ercolani G., Vespasiani Gentilucci U., Faloppi L. (2017). Interplay Between SIRT-3, Metabolism and Its Tumor Suppressor Role in Hepatocellular Carcinoma. Dig. Dis. Sci..

[180] Hu X., Wang D., Sun L., Gao Y., Zhou D., Tong X., Li J., Lin H., Qing Y., Du S. (2021). Disturbed mitochondrial acetylation in accordance with the availability of acetyl groups in hepatocellular carcinoma. Mitochondrion.

[181] Zhang C.Z., Liu L., Cai M., Pan Y., Fu J., Cao Y., Yun J. (2012). Low SIRT3 expression correlates with poor differentiation and unfavorable prognosis in primary hepatocellular carcinoma. PLoS ONE.

[182] Newman J.C., He W., Verdin E. (2012). Mitochondrial protein acylation and intermediary metabolism: Regulation by sirtuins and implications for metabolic disease. J. Biol. Chem..

[183] Zeng X., Wang N., Zhai H., Wang R., Wu J., Pu W. (2017). SIRT3 functions as a tumor suppressor in hepatocellular carcinoma. Tumour Biol..

[184] Song C.L., Tang H., Ran L.K., Ko B.C., Zhang Z.Z., Chen X., Ren J.H., Tao N.N., Li W.Y., Huang A.L. (2016). Sirtuin 3 inhibits hepatocellular carcinoma growth through the glycogen synthase kinase-3beta/BCL2-associated X protein-dependent apoptotic pathway. Oncogene.

[185] Zhang R., Wang C., Tian Y., Yao Y., Mao J., Wang H., Li Z., Xu Y., Ye M., Wang L. (2019). SIRT5 Promotes Hepatocellular Carcinoma Progression by Regulating Mitochondrial Apoptosis. J. Cancer.

[186] Huang Z., Zhao J., Deng W., Chen Y., Shang J., Song K., Zhang L., Wang C., Lu S., Yang X. (2018). Identification of a cellularly active SIRT6 allosteric activator. Nat. Chem. Biol..

[187] Lim J.H., Lee Y.M., Chun Y.S., Chen J., Kim J.E., Park J.W. (2010). Sirtuin 1 modulates cellular responses to hypoxia by deacetylating hypoxia-inducible factor 1alpha. Mol. Cell.

[188] Laemmle A., Lechleiter A., Roh V., Schwarz C., Portmann S., Furer C., Keogh A., Tschan M.P., Candinas D., Vorburger S.A. (2012). Inhibition of SIRT1 impairs the accumulation and transcriptional activity of HIF-1alpha protein under hypoxic conditions. PLoS ONE.

[189] Garcia-Rodriguez J.L., Barbier-Torres L., Fernandez-Alvarez S., Gutierrez-de Juan V., Monte M.J., Halilbasic E., Herranz D., Alvarez L., Aspichueta P., Marin J.J. (2014). SIRT1 controls liver regeneration by regulating bile acid metabolism through farnesoid X receptor and mammalian target of rapamycin signaling. Hepatology.

[190] Karbasforooshan H., Hayes A.W., Mohammadzadeh N., Zirak M.R., Karimi G. (2020). The possible role of Sirtuins and microRNAs in hepatocellular carcinoma therapy. Cell Cycle.

[191] Kim J.K., Noh J.H., Jung K.H., Eun J.W., Bae H.J., Kim M.G., Chang Y.G., Shen Q., Park W.S., Lee J.Y. (2013). Sirtuin7 oncogenic potential in human hepatocellular carcinoma and its regulation by the tumor suppressors MiR-125a-5p and MiR-125b. Hepatology.

[192] Zhao J., Wozniak A., Adams A., Cox J., Vittal A., Voss J., Bridges B., Weinman S.A., Li Z. (2019). SIRT7 regulates hepatocellular carcinoma response to therapy by altering the p53-dependent cell death pathway. J. Exp. Clin. Cancer Res..

[193] Jo H., Park Y., Kim T., Kim J., Lee J.S., Kim S.Y., Chung J.I., Ko H.Y., Pyun J.C., Kim K.S. (2020). Modulation of SIRT3 expression through CDK4/6 enhances the anti-cancer effect of sorafenib in hepatocellular carcinoma cells. BMC Cancer.

[194] Tao N.N., Zhou H.Z., Tang H., Cai X.F., Zhang W.L., Ren J.H., Zhou L., Chen X., Chen K., Li W.Y. (2016). Sirtuin 3 enhanced drug sensitivity of human hepatoma cells through glutathione S-transferase pi 1/JNK signaling pathway. Oncotarget.

[195] De Matteis S., Scarpi E., Granato A.M., Vespasiani-Gentilucci U., La Barba G., Foschi F.G., Bandini E., Ghetti M., Marisi G., Cravero P. (2019). Role of SIRT-3, p-mTOR and HIF-1alpha in Hepatocellular Carcinoma Patients Affected by Metabolic Dysfunctions and in Chronic Treatment with Metformin. Int. J. Mol. Sci..

[196] Wang R., Liu Y., Mi X., Chen Q., Jiang P., Hou J., Lin Y., Li S., Ji B., Fang Y. (2020). Sirt3 promotes hepatocellular carcinoma cells sensitivity to regorafenib through the acceleration of mitochondrial dysfunction. Arch. Biochem. Biophys..

[197] Xia Y.Q., Hua R.J., Juan C., Zhong Z.H., Tao C.S., Fang R., Lin H., Rui G., Yong C. (2018). SIRT6 Depletion Sensitizes Human Hepatoma Cells to Chemotherapeutics by Downregulating MDR1 Expression. Front. Pharm..

[198] Chen J., Zhang B., Wong N., Lo A.W., To K.F., Chan A.W., Ng M.H., Ho C.Y., Cheng S.H., Lai P.B. (2011). Sirtuin 1 is upregulated in a subset of hepatocellular carcinomas where it is essential for telomere maintenance and tumor cell growth. Cancer Res..

[199] Chen J., Chan A.W., To K.F., Chen W., Zhang Z., Ren J., Song C., Cheung Y.S., Lai P.B., Cheng S.H. (2013). SIRT2 overexpression in hepatocellular carcinoma mediates epithelial to mesenchymal transition by protein kinase B/glycogen synthase kinase-3beta/beta-catenin signaling. Hepatology.

[200] Nancy Han M., Rong Wei P. (2021). Age-Adjusted Death Rates for Alzheimer Disease Among Adults Aged ≤ 65 Years, by Sex—National Vital Statistics System, United States, 1999–2019. MMWR Morb. Mortal Wkly..

[201] Yen T.C., Chen Y.S., King K.L., Yeh S.H., Wei Y.H. (1989). Liver Mitochondrial Respiratory Functions Decline with Age. Biochem. Biophys. Res. Commun..

[202] Turrens J.F. (2003). Mitochondrial formation of reactive oxygen species. J. Physiol..

[203] Houtkooper R.H., Argmann C., Houten S.M., Canto C., Jeninga E.H., Andreux P.A., Thomas C., Doenlen R., Schoonjans K., Auwerx J. (2011). The metabolic footprint of aging in mice. Sci. Rep..

[204] Bradshaw P.C. (2021). Acetyl-CoA Metabolism and Histone Acetylation in the Regulation of Aging and Lifespan. Antioxidants.

[205] Wagner G.R., Payne R.M. (2011). Mitochondrial acetylation and diseases of aging. J. Aging Res..

[206] Baldensperger T., Eggen M., Kappen J., Winterhalter P.R., Pfirrmann T., Glomb M.A. (2020). Comprehensive analysis of posttranslational protein modifications in aging of subcellular compartments. Sci. Rep..

[207] Anderson K.A., Green M.F., Huynh F.K., Wagner G.R., Hirschey M.D. (2014). SnapShot: Mammalian Sirtuins. Cell.

[208] Brown K., Xie S., Qiu X., Mohrin M., Shin J., Liu Y., Zhang D., Scadden D.T., Chen D. (2013). SIRT3 reverses aging-associated degeneration. Cell Rep..

[209] Ansari A., Rahman M.S., Saha S.K., Saikot F.K., Deep A., Kim K.H. (2017). Function of the SIRT3 mitochondrial deacetylase in cellular physiology, cancer, and neurodegenerative disease. Aging Cell.

[210] Nahalkova J. (2022). Focus on Molecular Functions of Anti-Aging Deacetylase SIRT3. Biochemistry.

[211] Lee J.Y., Hong S.H. (2020). Hematopoietic Stem Cells and Their Roles in Tissue Regeneration. Int. J. Stem Cells.

[212] Roichman A., Elhanati S., Aon M.A., Abramovich I., Di Francesco A., Shahar Y., Avivi M.Y., Shurgi M., Rubinstein A., Wiesner Y. (2021). Restoration of energy homeostasis by SIRT6 extends healthy lifespan. Nat. Commun..

[213] Okabe K., Yaku K., Tobe K., Nakagawa T. (2019). Implications of altered NAD metabolism in metabolic disorders. J. Biomed. Sci..

[214] Imai S., Guarente L. (2014). NAD+ and sirtuins in aging and disease. Trends Cell Biol..

[215] Zhu X.H., Lu M., Lee B.Y., Ugurbil K., Chen W. (2015). In vivo NAD assay reveals the intracellular NAD contents and redox state in healthy human brain and their age dependences. Proc. Natl. Acad. Sci. USA.

[216] Luengo A., Li Z., Gui D.Y., Sullivan L.B., Zagorulya M., Do B.T., Ferreira R., Naamati A., Ali A., Lewis C.A. (2021). Increased demand for NAD(+) relative to ATP drives aerobic glycolysis. Mol. Cell.

[217] Giblin W., Skinner M.E., Lombard D.B. (2014). Sirtuins: Guardians of mammalian healthspan. Trends Genet..

[218] Mukherji A., Bailey S.M., Staels B., Baumert T.F. (2019). The circadian clock and liver function in health and disease. J. Hepatol..

[219] Sato S., Solanas G., Peixoto F.O., Bee L., Symeonidi A., Schmidt M.S., Brenner C., Masri S., Benitah S.A., Sassone-Corsi P. (2017). Circadian Reprogramming in the Liver Identifies Metabolic Pathways of Aging. Cell.

[220] Levine D.C., Hong H., Weidemann B.J., Ramsey K.M., Affinati A.H., Schmidt M.S., Cedernaes J., Omura C., Braun R., Lee C. (2020). NAD(+) Controls Circadian Reprogramming through PER2 Nuclear Translocation to Counter Aging. Mol. Cell.

[221] Milne J.C., Lambert P.D., Schenk S., Carney D.P., Smith J.J., Gagne D.J., Jin L., Boss O., Perni R.B., Vu C.B. (2007). Small molecule activators of SIRT1 as therapeutics for the treatment of type 2 diabetes. Nature.

[222] Mouchiroud L., Houtkooper R.H., Moullan N., Katsyuba E., Ryu D., Canto C., Mottis A., Jo Y.S., Viswanathan M., Schoonjans K. (2013). The NAD(+)/Sirtuin Pathway Modulates Longevity through Activation of Mitochondrial UPR and FOXO Signaling. Cell.

[223] Aman Y., Qiu Y., Tao J., Fang E.F. (2018). Therapeutic potential of boosting NAD+ in aging and age-related diseases. Transl. Med. Aging.

